# Sustainable hydrogel from biowaste: Synthesis and characterization of pectin and starch-based hydrogels derived from fruit peel

**DOI:** 10.1371/journal.pone.0354244

**Published:** 2026-07-21

**Authors:** Aneesa Rasheed Anwar, Roudha Sultan Almarri, Munawwar Ali Khan, Pramod Kumbhar

**Affiliations:** 1 Department of Environmental Sciences & Sustainability, College of Natural & Health Sciences, Zayed University, Dubai, United Arab Emirates; 2 Department of Environmental Sciences & Sustainability, College of Natural & Health Sciences, Zayed University, Abu Dhabi, United Arab Emirates; IGDTUW: Indira Gandhi Delhi Technical University for Women, INDIA

## Abstract

Arid regions such as the United Arab Emirates face persistent challenges in agricultural productivity due to water scarcity, poor soil quality, and the overuse of chemical fertilizers. As a sustainable alternative, biodegradable hydrogels offer promising solutions owing to their high swelling capacity, water retention ability, and controlled release of water and nutrients. This study explored the synthesis and characterization of eco-friendly hydrogels derived from pectin and starch extracted from orange and banana peels, respectively. These biopolymers are cross-linked using calcium and polyvinyl alcohol, forming hydrogels suitable for agricultural applications. The quality of the extracted biopolymers was assessed by analyzing moisture content, ash content, equivalent weight, solubility, and pectin methylation degree. The results confirmed high purity, with low ash contents of 2.16% (pectin) and 2.31% (starch), and moisture contents of 17.66% and 10%, respectively, indicating good stability and storage potential. The low methoxy pectin content resulted in a degree of methylation of 6.08%, which was favorable for gel formation. Structural and morphological characterization via FTIR, SEM, and SEM-EDX revealed well-defined functional groups, a highly interconnected porous morphology, and structural integrity of the hydrogels even after 20 days of biodegradation. Swelling and pot experiments demonstrated that both hydrogels effectively retained soil moisture for up to 12 days, with swelling ratios ranging from 300% to 350% of their original weight. Biodegradation studies indicated that starch-based hydrogels degraded faster (78.76% weight loss) than pectin-based hydrogels did (67%), likely due to differences in pore size and structural compactness. Overall, the results validate the feasibility of producing sustainable, biodegradable hydrogels from fruit peel waste, with significant potential for water conservation and soil enhancement in arid agricultural systems.

## 1. Introduction

Hydrogels are three-dimensional, chemically cross-linked polymeric networks that can absorb and retain large quantities of water within their structure. These materials have attracted increasing attention for applications in agriculture, biomedicine, and environmental remediation due to their tunable physicochemical properties. Natural polymer-based hydrogels, in particular, have emerged as promising alternatives to their synthetic counterparts because of their biodegradability, biocompatibility, low toxicity, and sustainability. Among these, pectin and starch are widely available, plant-derived polysaccharides that offer low-cost and eco-friendly options for hydrogel synthesis [1,2]. These biopolymers have been studied for their potential to increase soil water retention, particularly under drought-prone conditions, contributing to climate-resilient agriculture [3,4]

Pectin is a naturally occurring anionic polysaccharide that is abundant in citrus fruit and apple pomace peels and constitutes approximately 50–60% of fruit processing waste [5] Rich in carboxylic functional groups, pectin serves as an effective natural substitute for acrylic acid-based superabsorbents, enabling the development of hydrogels with high swelling and ionic adsorption capacities [6,7] Studies have demonstrated the efficiency of pectin-based hydrogels in terms of water purification, nutrient release, and especially heavy metal ion removal from soil and wastewater systems [8, 9]. Moreover, modifying pectin with agents such as chitosan has been shown to significantly increase its water absorption performance [2]. These features position pectin hydrogels as multifunctional materials for environmental and agricultural use.

Starch, another abundant and renewable biopolymer found in crops such as corn, rice, potato, and banana, is similarly suitable for hydrogel synthesis [10, 11] It has excellent hydration and biodegradability properties and has been applied in the removal of toxic organic compounds, including dyes, pesticides, and heavy metals [12] However, the native form of starch suffers from weak mechanical stability due to its reliance on hydrogen bonding, which limits its structural integrity upon swelling. This issue can be mitigated by introducing cross-linking agents such as polyvinyl alcohol (PVA), a biodegradable, hydrophilic synthetic polymer that improves the physical stability and swelling behavior of starch-based hydrogels [13,14]. Previous work has successfully demonstrated the use of PVA-starch composites in applications such as controlled nutrient delivery [15]

Despite these advances, a notable gap persists in the literature regarding the direct utilization of pectin and starch extracted from real-world agricultural waste, particularly fruit peels, for hydrogel synthesis. Most studies rely on commercially purified biopolymers, which increase costs and limit the process’s sustainability. In this context, the present study focuses on the development of pectin and starch hydrogels derived from fruit-processing biowaste, prioritizing resource valorization rather than developing a new method for crosslinking. So this approach aligns with principles of green chemistry and a circular bioeconomy by reducing waste and promoting the use of renewable raw materials. This approach not only adds value to agri-food waste streams but also helps reduce the environmental burdens associated with waste disposal. Despite extensive literature on hydrogel synthesis from biopolymers, there is limited work on using food waste sources specific to the UAE or the Gulf region, where high temperatures and soil salinity demand regionally validated solutions. This study addresses this gap by using locally sourced fruit peels from Dubai’s food waste stream, and it provides baseline material characterization and comparisons to establish studies that lead to future Gulf region applications.

Fruit peels, including banana and citrus, account for 25–30% of fruit and vegetable processing waste. This highlights the need to use fruit waste to develop eco-friendly, value-added products. The present study focuses on utilizing the fruit waste, which is abundant in natural polymers that can be transformed into hydrogels with beneficial properties for agricultural applications, such as soil moisture retention, as well as biodegradability, which reduces product retention in soil post-use. The ability of the developed hydrogel to adsorb metal ions, as evidenced by EDX analysis, is an added functional property. At this point, we did not focus on the field trial; our aim was to validate and assess the feasibility of generating hydrogels and utilizing waste materials. Detailed chemical characterization of the extracted material, as well as the pot experiments, is under consideration.

## 2. Materials and methods

### 2.1. Materials

No specific permits or ethical approvals were required for this study, as all materials (fruit peels) were obtained from publicly accessible sources, including local cafeterias and markets, and no protected or regulated biological materials were used. Orange peels were collected from local cafeterias in Dubai, whereas banana peels were obtained from unripe bananas (stage 1–2) purchased at a local vegetable market; the bananas were subsequently used as food. The collected peels were immediately stored at 4 °C until processing. All chemicals were used as received without further purification. Polyvinyl alcohol (PVA, Mw 30,000), citric acid, and sodium metabisulfite were obtained from S D Fine-Chem Limited, India. Sodium hydroxide was procured from BioWORLD, USA, and calcium chloride was obtained from Sigma‒Aldrich, Japan. Distilled water was used throughout the study.

### 2.2. Extraction of pectin from orange peels

The outer flavedo of the orange peels was manually removed via a fruit peeler. The inner white albedo layer was retained, washed thoroughly, cut into small pieces, and oven-dried at 70 °C for 3 days. The dried peels were ground into a powder in a mill and stored in sealed Ziploc bags. Pectin was extracted via acid hydrolysis [16] with slight modifications. The peel powder was mixed with a 1:30 (w/v) ratio of citric acid solution (pH 1.5) and heated at 90 °C for 80 minutes. The mixture was filtered through cheesecloth, and the filtrate was treated with twice its volume of ice-cold ethanol (96%) to precipitate the pectin. The solution was refrigerated at 4 °C for 18 h. The precipitate was recovered by centrifugation at 4000 rpm for 20 min, washed with distilled water, oven-dried at 60 °C, and ground in a laboratory mill. The extraction was repeated in triplicate for reproducibility.

### 2.3. Extraction of starch from banana peels

Banana peels were washed in tap water for 3 times, chopped using a knife and soaked in 1% (w/v) sodium metabisulfite solution for 15 minutes to prevent oxidation [10] and then oven-dried at 70 °C until a constant weight was achieved. The dried material was powdered and stored. For extraction, the peel powder was homogenized in 0.2% sodium metabisulfite solution (1:10 w/v) for 1 h. The mixture was squeezed through cheesecloth, and the filtrate was left to settle for 24 h. The sedimented starch was collected by decanting and washed with deionized water. After drying at 60 °C for 24 h, the starch was powdered using a mortar and pestle and stored in airtight containers. The yield (%) of extracted pectin and starch was calculated via the following equation (1):


$$\begin{array}{c} Yeild\;(\%)=\;\frac{Weight\;of\;extracted\;material\;(g)}{Weight\;of\;powder\;taken\;for\;extraction}\;\times \text{1}\text{0}\text{0} \end{array}$$ (1)

### 2.4. Synthesis of the pectin hydrogel

Pectin hydrogels were synthesized using CaCl_2_ as a cross-linking agent [6]. A 5% pectin solution was prepared in distilled water and stirred until semitransparent. Then, 10 mL of 10% CaCl_2_ solution was added dropwise under vigorous stirring. The solution was kept at room temperature for 24 h. Afterward, 10% NaOH and 10 mL of methanol were added sequentially, and the mixture was left for 12 h to induce gelation. The resulting gel was filtered through a Buchner funnel layered with filter paper, transferred to Petri dishes, and dried at 40 °C for 48 h.

### 2.5. Synthesis of starch hydrogel

Starch hydrogels were prepared using PVA as a primary cross-linking agent. Starch (4 g) was dispersed in 30 mL of distilled water, heated to 90 °C for 25 minutes with constant stirring for thickening, and then cooled to 50 °C. To this a 10% PVA solution was added while stirring, followed by the addition of a few drops of 2 M NaOH to induce gelation [17] by adjusting the pH. The mixture was poured into weighing boats and freeze-dried (SP Scientific) at −20 °C and 500 Pa for 6 h.

### 2.6. Characterization of extracted pectin and starch

#### 2.6.1. Moisture content of pectin and starch.

Approximately 1 g of each sample was placed in a preweighed crucible, dried at 100 °C for 4 h in a hot air oven, cooled in a desiccator, and reweighed. The process continued until a constant weight was achieved [18]. The moisture content was calculated via the following equation (2):


$$\begin{array}{c} Moisture\;(\%)=\;\frac{W_{\text{2}}\;(g)-W_{\text{3}}\;(g)}{W_{\text{2}\;}(g)-W_{\text{1}\;}(g)}\times \text{1}\text{0}\text{0}. \end{array}$$ (2)

*W*_*1*_ = the weight of the empty crucible, *W*_*2*_ = the weight of the crucible and powder, and *W*_*3*_ = the total weight of the dried powder and crucible.

#### 2.6.2. Ash contents of pectin and starch.

The ash content was determined by incinerating 1 g of the sample in a dry, pre-weighed crucible in a muffle furnace at 600 °C for 5 h. Once done, the crucible was taken out of the furnace and kept in a desiccator to cool down. The crucible was reweighed, and this process was continued until a constant weight was achieved. The ash content was calculated via the following equation (3):


$$\begin{array}{c} Ash\;(\%)=\;\frac{W_{\text{2}}\;(g)-W_{\text{1}}\;(g)}{W\;(g)}\times \text{1}\text{0}\text{0} \end{array}$$ (3)

*W* = weight of the pectin sample, *W*_*1*_ = weight of the dish, and *W*_*2*_ = final weight of the dish and ash.

#### 2.6.3. Equivalent weight of pectin.

To determine the equivalent weight of pectin, 0.25 g of the sample was moistened with 5 mL ethanol and then dissolved in 100 mL of warm distilled water containing 1 g NaCl. Phenolphthalein was added, and the solution was titrated with 0.1 N NaOH [19]. The equivalent weight was calculated via the following equation (4):


$$\begin{array}{c} Equivalent\;weight=\;\frac{Weight\;of\;pectin(g)\;\times \;\text{1}\text{0}\text{0}\text{0}}{Volume\;of\;alkali\;\times \;Normality\;of\;alkali} \end{array}$$ (4)

#### 2.6.4. Methoxyl content and degree of esterification (DE).

The neutral solution from the equivalent weight test was treated with 25 mL of 0.25 M NaOH, followed by 25 mL of 0.25 M HCl. Titration with 0.1 N NaOH was performed using phenolphthalein as an indicator [19]. The methoxyl content was calculated via the following equation (5):


$$\begin{array}{c} Methoxyl\;content=\;\frac{Volume\;of\;alkali(mL)\times Normality\;of\;alkali\times \text{3}\text{1}\times \text{1}\text{0}\text{0}}{weight\;of\;sample\;(mg)} \end{array}$$ (5)

The DE of the pectin was determined using the standard titrimetric method, using the following equation (6)


$$\begin{array}{c} Degree\;of\;Esterification\;(\%)=\frac{V_{\text{2}}}{V_{\text{1}}+\;V_{\text{2}}}\times \text{1}\text{0}\text{0}. \end{array}$$ (6)

### 2.7. Swelling studies

The swelling ratio was measured using filtration [20]. Approximately 0.1 g of dried hydrogel was placed in a tea bag and immersed in 100 mL of water at room temperature. At regular intervals, the samples were removed, blotted with absorbent filter paper for 10 sec under minimal and uniform pressure to gently remove surface-adhered water without affecting the internal water content of the hydrogel, and weighed. The swelling ratio was calculated via the following equation (7):


$$\begin{array}{c} Swelling\;ratio\;(\%)=\;\frac{W_{\text{2}}-W_{\text{1}}-W_{\text{0}}}{W_{\text{0}}} \end{array}$$ (7)

*W*_*0*_ = initial weight of the powder*, W*_*1*_ = weight of the empty tea bag, *and W*_*2*_ = weight of the tea bag and sample after a certain time interval*.*

### 2.8. Water retention in soil

Hydrogels (0.34 g) were mixed with 170 g of commercial potting soil (pH 6.5, EC 1.5 dS m^-1^, soil texture: loamy, well aerated, moisture percentage- 47%) in plastic pots and watered with 40 mL of distilled water. The control pots contained only soil. All experiments were performed in triplicate. Pots were weighed every two days for 14 days at room temperature. Water retention was calculated via the equation (8)


$$\begin{array}{c} Retention\;(\%)=\frac{R_{\text{0}\;}(g)-R_{t}(g)}{R_{\text{0}}(g)-R_{i}(g)}\times \;\text{1}\text{0}\text{0} \end{array}$$ (8)

where *R (%)* = percentage of retained water, *R*_*i =*_ initial weight before adding water, *R*_*t =*_ weight at time *t,* and *R*_*0*_ = weight at time zero (after draining the added water).

### 2.9. Hydrogel biodegradability test

The hydrogels (0.3 g) were buried 5 cm deep in 12 cm soil pots and maintained at room temperature. At 5-day intervals for up to 20 days, samples were retrieved, cleaned with ethanol and water, dried, and weighed. The biodegradability was calculated via the following equation (9):


$$\begin{array}{c} Biodegradability(\%)=\;\frac{M_{\text{0}}\;(g)-M_{t}(g)}{M_{\text{0}}\;(g)} \end{array}$$ (9)

where *M*_*0*=_ the initial weight of the hydrogels, and *M*_*t*_ is the weight at time t.

### 2.10. Surface morphology and elemental analysis

Scanning electron microscopy (TESCAN VEGA-3) was used to analyze the surface morphology. The samples were gold-coated for 120 s at 20 mbar and scanned at 10 kV. EDX (energy-dispersive X-ray spectroscopy) analysis was used for elemental profiling. The day 0 samples were directly imaged; the day 20 biodegraded samples were cleaned, oven-dried, and analyzed similarly.

### 2.11. Fourier transform infrared spectroscopy

Fourier transform infrared spectroscopy analysis (Agilent Cary 630) was performed on lyophilized hydrogel samples (freeze-dried at −60 °C for 12 h). To assess changes after biodegradation, the soil-buried samples were recovered after 20 days, cleaned, dried, and scanned in the range of 4000–500 cm^‒1^ [21].

### 2.12. Statistical analysis

Statistical analysis was performed using two- way ANOVA followed by Tukey’s post hoc analysis. Different letters indicate statistically significant differences between groups at p < 0.05. All experiments were conducted in triplicate and presented as mean ± standard deviation. Error bars in all figures represent standard deviation.

## 3. Results and discussion

### 3.1. Yields of pectin and starch from raw materials

The extraction yields of pectin and starch from orange and banana peels are summarized in Table 1. The yield of pectin from orange peels was 38.82 ± 0.51%, which is consistent with previously reported values ranging from 12.93–31.21% using citrus-based sources and acid extraction methods [22,23]. A previous study [24] reported yields of 21–30% from Egyptian orange peel. Studies suggest [25] that citric acid extraction significantly enhances the pectin yield. Similarly, apple pomace yielded 56.6% pectin when extracted with citric acid, compared to ~12% with hydrochloric or sulphuric acid [26]. The low pH (1.5) used in the present study likely facilitated the effective hydrolysis of glycosidic linkages, releasing more pectin. Here, the pectin was extracted at fixed conditions to serve as a baseline for hydrogel preparation. It has been previously reported that the extraction of pectin from grapefruit, citron, and pomelo peels resulted in higher yields when an aqueous solution with a pH of 1.5 was used, compared to extractions performed within a wider range of 1.5–4.2 [27]. While preliminary trials validated the selected experimental conditions, further statistical optimization and scale-up studies have to be designed to better adapt large-scale applications.

**Table 1:** Characteristics of pectin and starch extracted from orange and banana peels, respectively.

Raw material	% yield	Moisture content (%)	Ash content (%)
Orange Peel	38.82 ± 0.51	17.66 ± 0.57	2.16 ± 0.17
Banana Peel	13.63 ± 0.80	10 ± 0.5	2.31 ± 0.56

The yield of starch from banana peels was 13.63 ± 0.80%, similar to previously reported values [16,28] with a yield of around 10–13%. However, higher yields of up to 22–48% have been reported [29,30]. This variation can be attributed to the maturity of the banana, where the starch content decreases as the fruit ripens [31], as well as to various environmental differences.

### 3.2. Moisture and ash content

Moisture content is an important parameter influencing storage stability. The pectin extracted from orange peels had a moisture content of 17.66 ± 0.57%, which was slightly higher than literature values of 10.07% [32] and 15.03% [33] (Susanti et al., 2021). This could be due to post-drying absorption during refrigerated storage. Similar results were reported [34]in orange peel with moisture levels of 13.39% for sulfuric acid-extracted pectin and 11.49% for citric acid-extracted pectin. A moisture content of 10.00 ± 0.50% was recorded for banana peel starch, which aligns with values reported for plantain and Agung banana peels [35,36]. The ideal moisture content for starch powder should be < 20% to improve storage stability and prevent microbial contamination [Hadisoewignyo et al., 2017].

The ash content, indicative of inorganic matter, was 2.16 ± 0.17% for pectin and 2.31 ± 0.56% for starch. These values fall within the range of previous studies [34,37]. A low ash content generally correlates with higher pectin purity and better gel-forming properties [38]. For starch, ash values reported in the literature range from 0.98% to 7.85%, depending on the source [35,39,40].

### 3.3. Equivalent weight of pectin

The equivalent weight, which represents the free galacturonic acid content, was determined to be 102.45 mg/mol. This value aligns with reports for citrus and tangerine peels, which range from 86.87–116.78 mg/mol [41, 42]. A higher value of 312.5 mg/mol has been reported from sweet lemon peel [43]. The equivalent weight is influenced by the extraction pH, temperature, and acid type, with a lower pH often yielding partially degraded polymers and thus a lower equivalent weight [44,45].

### 3.4. Degree of methylation and esterification in pectin

The methoxyl content of the extracted pectin was 6.08%, which was classified as low-methoxyl pectin (<7%). This property is beneficial for ionic gelation in the presence of calcium [46, 47]. Similar values were reported for apple pomace and citrus sources [48, 49]. A lower degree of methylation provides more free carboxyl groups, enhancing the gel strength and cross-linking efficiency [44]. The degree of esterification (DE) of the extracted pectin was 16.7%. Pectin with low DE value is known to form gels by ionic crosslinking with divalent cations such as Ca^2+^ through the eggbox mechanism, which enhances hydrogel formation and controlled release application [50]. The low DE observed in this study may be attributed to partial de-esterification during organic acid extraction. Similar trends have been reported in previous studies [51].

### 3.5. Swelling capacity of the hydrogels

**Fig 1:**
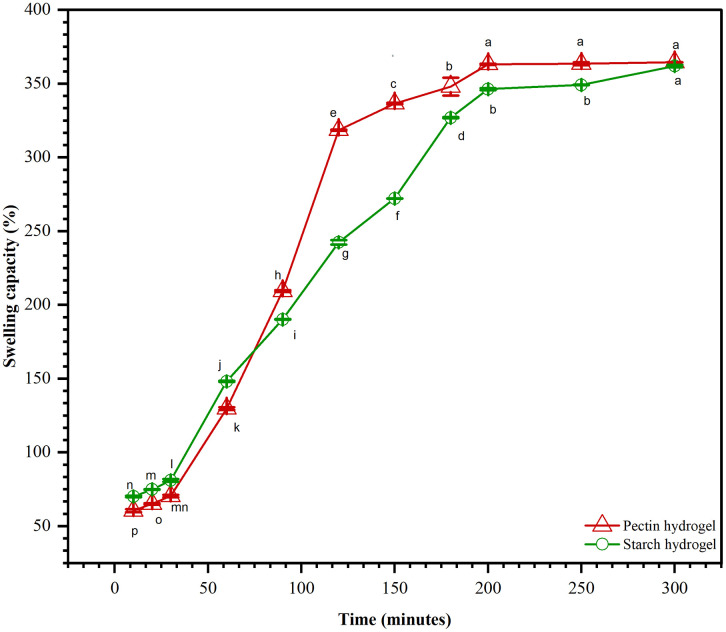
Swelling capacity of pectin and starch hydrogels in distilled water over time. Each value indicates the mean of triplicate values. The bars represent the mean standard deviation.

The swelling capacity of starch and pectin hydrogels significantly increased over time. The swelling behavior varied with both hydrogel composition and time; a significant interaction between time and hydrogel type was observed (p < 0.0001). Swelling studies revealed a three-phase swelling profile for both hydrogels (Fig 1): initial uptake (0–30 min), rapid absorption (60–180 min), and a plateau phase (180–350 min), similar to the patterns reported by [52,53]. At the equilibrium time of 250 min, the pectin hydrogel achieved a maximum swelling ratio of 363.44a ± 1.02%, whereas the starch hydrogel reached 349.09b ± 0.20%.

The high swelling capacity of the pectin hydrogel is attributed to its porous morphology and efficient cross-linking with Ca² ⁺ ions, both of which enhance water retention. In contrast, starch hydrogels cross-linked with PVA also demonstrated excellent performance due to PVA’s hydrophilic nature and its ability to form loose, interpenetrating networks [54]. Previous studies have confirmed that cross-linking significantly improves hydrogel integrity and swelling performance [55, 56]. This experiment provides a controlled baseline for assessing the intrinsic swelling capacity of hydrogels in distilled water. The presence of dissolved salts and ions can significantly influence hydrogel swelling behavior by affecting osmotic pressure and polymer network interactions, so the performance of hydrogels under these conditions needs to be studied to simulate real soil environments. The swelling behavior observed in these results shows that the hydrogel network readily absorbs water, due to the abundant hydrophilic functional groups in the polymers and the crosslinking network. Natural polymers based on pectin and starch exhibit enhanced swelling capacities, as seen in fruit waste composites, reporting swelling values of ~300–400% in the sandy soil system [16]. Biodegradable pectin-starch systems with moderate swelling have been reported [57]. Compared to this study, the current hydrogel shows a slightly higher percentage when adapted to fruit waste streams relevant to the Gulf region.

### 3.6. Water retention studies

**Fig 2:**
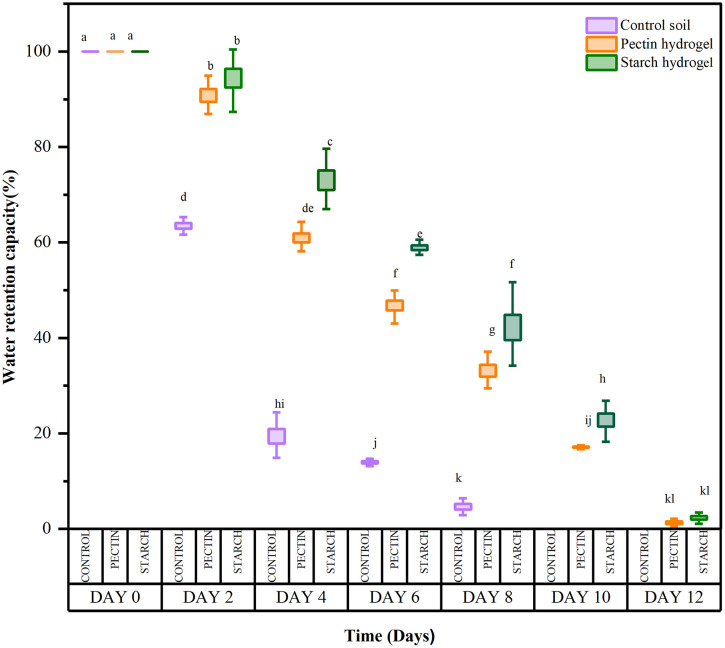
Water retention capacity of pectin and starch hydrogels- amended soil compared with control soil over 20 days. Each value indicates the mean of triplicate values. The bars represent the mean standard deviation.

The study demonstrated that both time and hydrogel type significantly influenced water retention, indicating the effectiveness of treatment over time. Hydrogels significantly improved soil water retention (Fig 2). On day 2, water retention in starch- and pectin-amended soils was 93.89a ± 2.18% and 90.92b ± 1.34%, respectively, whereas it was 63.47d ± 0.61% in the control. By day 6, the soil amended with starch-based hydrogel retained 58.99e ± 0.53% of water content, whereas the soil amended with pectin retained 46.48f ± 1.15%. Even after 8 days, the moisture content of the hydrogel-treated soils remained greater than 30%, whereas that of the control decreased to 4.65%. The pairwise comparisons using Tukey’s test showed that among the treatments, starch had the highest water retention, followed by pectin, which is attributed to its hydrophilic polymeric structure that can retain water. Both were significantly higher than the control (p < 0.05). These results align with studies showing improvements in retention with biopolymer hydrogels [57, 58]. The hydrogel matrix slowly releases stored water as the soil dries, reducing evaporation and deep percolation losses [4]. The structure allows for the retention of loosely bound, fully bound, and half-bound water [57,59], making this a potential material suitable for arid and semiarid conditions. The direct effect of the hydrogel on plant growth needs validation through a pot experiment and field trials that are similar to the UAE agricultural needs and arid conditions.

### 3.7. Biodegradation of hydrogels

**Fig 3:**
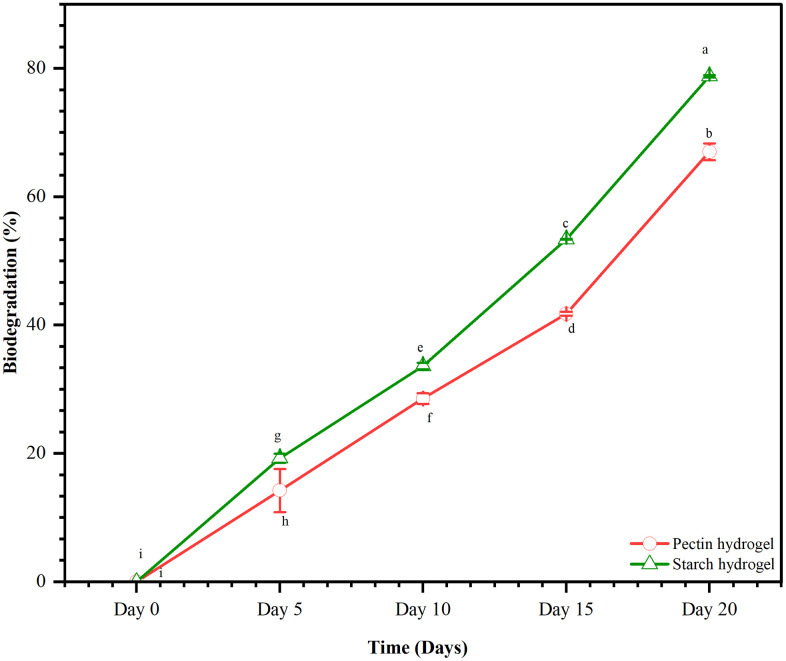
Biodegradation of pectin and starch hydrogels buried in soil over 20 days. Each value indicates the mean of triplicate values. The bars represent the mean and standard deviation.

Soil burial tests (Fig 3) revealed increasing degradation of both materials over time. The percentage of biodegradation (p < 0.0001) was significantly influenced by both time (days) and hydrogel type. By day 20, the starch hydrogel had degraded 78.76 ± 0.16%, while the pectin hydrogel had degraded 67.00 ± 1.28%. The post hoc analysis using Tukey’s test confirmed a significant difference among treatment – time combinations, with each time point forming statistically distinct groups. The faster degradation of the starch hydrogel is attributed to microbial and enzymatic degradation of PVA, as well as to its larger pore size [60]. In contrast, the pectin hydrogel rich in galacturonic acid units, along with the cross-linking, reduces the microbial effect and results in slower degradation. Previous studies have reported that starch-based systems degrade faster, so they can be blended with polymers to improve degradation rates [61]. A study on a starch/PVA blend reported that this combination is efficiently biodegradable [62]. From the methylation test, it is evident that the extracted pectin is low methoxyl pectin, which forms a stable gel; this, along with calcium chloride crosslinking, supports controlled degradation as environmental conditions change [63]. Biodegradable materials such as these are essential in sustainable agriculture to avoid post-functional soil accumulation [64]. Our results are consistent with findings that natural hydrogels degrade faster than their synthetic counterparts [65,66]. While the hydrogels exhibited promising biodegradation in potting mix, their degradation behavior in saline soil and under high-temperature conditions may differ; this highlights the need for further testing under field conditions to standardize the time required for complete biodegradation of the polymer. Compared with synthetic hydrogels, most exhibit very high swelling capacity and mechanical strength, but their persistence in soil will have a long-term environmental impact. In contrast, natural polymer-based hydrogels offer significant advantages in terms of biodegradability and ecological compatibility. In this study, a commercial hydrogel is not used as a control; the observed swelling and biodegradation percentages are consistent with previously reported values for polysaccharide-based hydrogels [67].

### 3.8. Morphological analysis

#### 3.8.1. Scanning electron microscopy.

**Fig 4:**
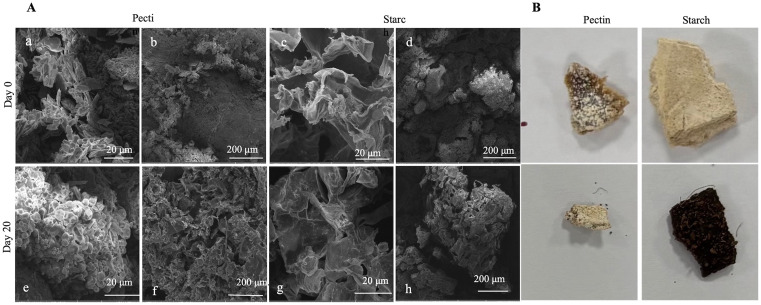
A. SEM image of pectin and starch hydrogel at 20X and 200X magnification. (a–d) Day 0: pectin and starch hydrogels; (e–h) day 20: pectin and starch hydrogels. Fig 4.B. Changes in morphology before and after degradation. a, b Pectin content after 0 and 20 days. c, d Starch hydrogel at 0 and 20 days.

SEM analysis revealed the surface morphology of the pectin- and starch-based hydrogels before and after 20 days of soil burial. The Day 0 images revealed a highly porous, interconnected network, characteristic of superabsorbent materials [68]. This structure enhances the hydrogel’s capacity for water absorption and retention. For the pectin hydrogel, the initial surface appeared smooth and dense, whereas the day 20 images revealed a rough, flaky morphology, indicating ongoing degradation (Fig 4. A. a–b). This transformation likely results from microbial activity and moisture-induced breakdown of cross-linking bonds, particularly between pectin and CaCl_2_ [21]. The starch hydrogel exhibited a lamellar and layered structure on Day 0 (Fig 4.B), which was conducive to high water uptake. After 20 days, the hydrogel showed signs of shrinkage and surface roughening with a color change from off-white to black. This suggests structural decomposition and microbial colonization. The pore size and distribution affect degradation; polymers with larger pores degrade more rapidly, as confirmed by previous studies [69,70]

#### 3.8.2. EDX analysis.

**Fig 5:**
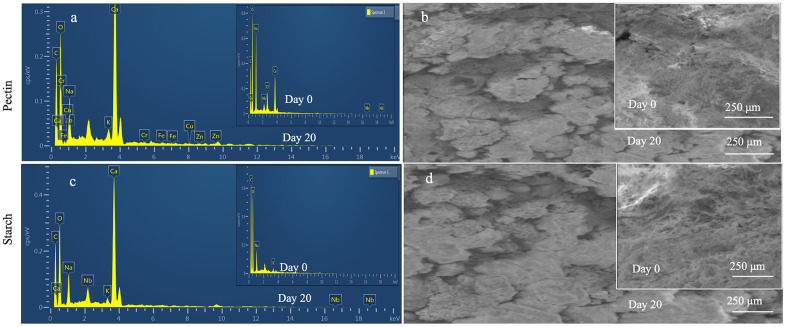
EDX spectra of (a) pectin and (c)starch hydrogels before (Day 0) and after (Day 20) soil burial. Pectin (b) and starch (d) hydrogels before and after soil burial.

EDX analysis revealed the presence of metal ions on the hydrogel after soil burial (Fig 5), suggesting the possible adsorption or interaction with soil components. For the pectin hydrogel on Day 0, the predominant elements were oxygen (49.29%) and carbon (25.71%), representing the organic backbone, as the orange peels in the hydrogel are rich in pectin, cellulose, and hemicellulose [71]. After 20 days of soil incubation, a decrease in oxygen content (44.96%) and an increase in carbon content (29.26%) were observed, indicating partial mineralization. Elements such as sodium (14.85%), calcium (6.44%), and chlorine (2.35%) confirmed the integration of CaCl_2_ and NaOH during cross-linking.

The decrease in sodium content (to 5.24%) and increase in calcium content (to 19.12%) suggest leaching and ion exchange. Before burial, the spectra predominantly showed elements corresponding to the polymeric network. After 20 days, the emergence of Fe, Cr, Cu, and Zn was detected. This elemental profile suggests an interaction between the hydrogel surface and the surrounding soil. The starch hydrogel showed high initial levels of carbon (50.03%) and oxygen (45.62%), typical of polysaccharides. Minor elements such as sodium (3.82%) and potassium (0.52%) were also present. After degradation, the carbon content decreased to 37.66%, the oxygen content decreased to 41.27%, and the calcium content increased to 14.56%, indicating mineral uptake. Notably, niobium (2.28%) and residual chlorine were observed, indicating minor elemental transfer during burial. However, it should be noted that EDX provides qualitative evidence and does not quantify adsorption capacity. Further studies on adsorption kinetics and capacity determination are required to fully assess the performance of the developed hydrogel across different salinity levels.

#### 3.8.3. FTIR analysis of pectin and starch hydrogels.

**Fig 6:**
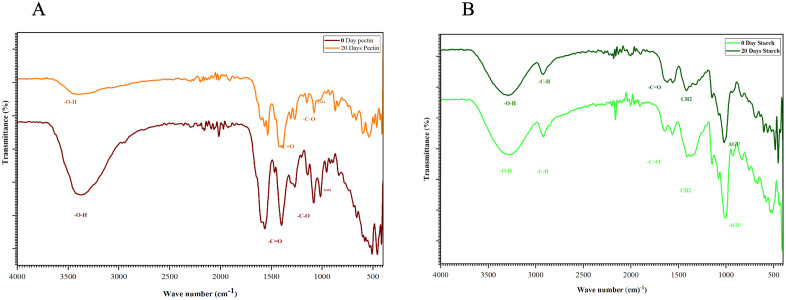
FTIR images of the (A) pectin and (B) starch hydrogels showing the change in functional groups after 20 days of biodegradation compared to 0 days.

FTIR spectroscopy revealed the functional groups present in the hydrogels and tracked their changes following soil degradation (Fig 6). For the pectin hydrogel, the broad peak at ~3249 cm^‒1^ corresponds to O–H stretching. The peak at 1579 cm^‒1^ is associated with carboxylate (COO⁻) groups, whereas the peak at 1403 cm^‒1^ indicates C–H bending. Peaks at 1244 cm^‒1^ (C–O), 1018 cm^‒1^ (galacturonic acid), and 895–920 cm^‒1^ reflect the α- and β-configurations of pectin linkages [72,73]. In the starch hydrogel, the broad peak at ~3342 cm^‒1^ corresponds to O–H stretching vibrations of carbohydrates. The FTIR spectrum shows a characteristic peak at 2927 cm^‒1^ corresponding to aliphatic C-H stretching. The peaks at 1655 cm^‒1^ and 1419 cm^‒1^ correspond to C–O bending and CH_2_ scissoring, respectively. Additional features include CH_2_ shear vibration at 1394 cm ⁻ ¹, CO stretching at 1153 cm^‒1^, and an anhydrous glucose ring (C–O–C) at 958 cm^‒1^ [74]. After biodegradation, a reduction in the intensity of O-H and carboxyl-related peaks was observed, indicating their degradation as hydroxyl and carboxyl groups are more susceptible to enzymatic degradation. The breakdown of the polysaccharide backbone is indicated by the decrease in peaks related to glycosidic bonds. The slow breakdown of the hydrogel structural network is confirmed by the weight loss observed during the soil burial test. The -OH and -COO^-^ groups are hydrophilic in nature and play a crucial part in water absorption, which is present in both starch and pectin hydrogel. Reduction in these functional groups after biodegradation likely contributes to reduced water retention capacity, highlighting their importance in maintaining hydrogel functionality [75–77]. In the pectin hydrogel, comparatively higher swelling was observed; this can be attributed to the availability of carboxyl groups, which enhance water uptake and osmotic pressure [78]. Although the pectin hydrogel showed a higher swelling ratio, the water retention of both hydrogels remained comparable. This indicates that swelling alone does not determine water retention capacity. SEM imaging reveals a porous, interconnected network that supports water retention via capillary forces within the matrix. The internal structure of the hydrogel and its interaction with the surrounding soil, supported by hydrogen bonding and capillary forces along with bound and free water, are the involved factors [79,80]. The combined SEM, FTIR, and functional analyses show that overall hydrogel function is dependent on both functionality and physical structure.

#### 3.8.4. Visual observations.

After 20 days of soil burial, visual and structural changes were evident. Pectin hydrogels change from bright orange to pale yellow due to the degradation of natural pigments [71]. The surface roughness and reduced thickness suggest cross-link loss. The starch hydrogels turned black with uneven surfaces. Increased surface porosity and embedded soil particles were evident, which is consistent with microbial degradation and pH changes [72]. These visual changes indicate interaction with the soil environment, but they do not provide definitive evidence of structural integrity or degradation behavior. To support this, the quantitative biodegradation data can be incorporated. The results show an increase in degradation for both systems, with two-way ANOVA confirming the influence of time and hydrogels on degradation (p < 0.0001). Notably, the starch-based hydrogel (78.76%) exhibited a higher degradation rate than the pectin hydrogel (67%). These indicate that the starch hydrogel degrades more rapidly, suggesting lower network stability under controlled conditions. SEM provides the overall structural morphology of the hydrogel, but qualitative analysis of pore size distribution and deeper surface area studies have to be considered in the future.

Such disintegration patterns were also observed in biodegradable guar gum and cellulose-based hydrogels [73], confirming that these bioderived hydrogels break down efficiently, leaving minimal residue. Although this study demonstrated the potential of pectin- and starch-based hydrogels from fruit waste, several limitations should be acknowledged. First, the experiments were conducted under controlled laboratory conditions with a constant temperature and a single soil type. Given Dubai’s extreme temperature fluctuations and diverse soil compositions, future research should evaluate hydrogel behavior under a wider range of climatic and soil conditions. Moreover, only orange and banana peels have been explored as sources of pectin and starch. Dubai’s food waste stream includes mangoes, papayas, watermelons, and other tropical fruits rich in biopolymers. The composition of fruit peel-derived polymer may vary with geographical and seasonal factors, which will affect the reproducibility. Large-scale extraction of raw materials and hydrogel synthesis may be challenging due to both cost and process efficiency. In terms of practical implementation, these aspects should be addressed. Detailed structural characterization of pectin (degree of polymerization and molecular weight distribution) and starch (gelatinization temperature and crystallinity) was not performed. The hydrogel characteristics, such as gel network structure and crosslink density, were not analyzed. Future work should address these aspects. While the characterization results were promising, it should be noted that the swelling percentage was evaluated using distilled water; swelling kinetics modeling, including the determination of the diffusion mechanism and swelling half-time, was not performed. Future studies should investigate the behavior of these hydrogels under varying salt concentrations to properly assess their field applicability. Although our study demonstrates improved water retention under controlled conditions, parameters such as germination, yield, and biomass have not yet been evaluated to assess the impact of the developed hydrogels on agricultural efficiency. Further experiments should be conducted in pots and in field trials, especially in an arid environment. Even though the biodegradation study results are promising, to further assess the adaptability of hydrogels to biodegradation under real UAE weather and soil conditions, a long-term evaluation is necessary under representative arid soil conditions. Further studies involving controlled adsorption experiments with known metal concentration and quantitative analysis are required to confirm and quantify metal uptake. The recovery of absorbed metal ions has to be studied. In addition, assessing residual chemicals after application and microbial contaminants before field application will be essential. Expanding the experimental matrix in this manner would significantly enhance the applicability and impact of this work in real-world agricultural settings.

## 4. Conclusion

This study successfully demonstrated the synthesis and characterization of sustainable hydrogels derived from fruit peel waste, specifically pectin from orange peels and starch from banana peels. The initial hypothesis that these biowaste-derived polymers could yield effective, biodegradable hydrogels for soil moisture retention and possible environmental remediation was supported by comprehensive experimental evidence. Characterization through SEM, EDX, and FTIR confirmed the integrity and functionality of the hydrogel networks. Pectin-based hydrogels displayed superior swelling behavior, absorbing up to 363.44% of their dry weight in water, whereas starch-based hydrogels provided better degradation rates. In practical applications, starch hydrogels maintain soil moisture for up to 10 days, and both materials degrade safely over a 20-day period in a controlled environment, indicating their environmental compatibility. Importantly, EDX analysis revealed the hydrogels’ capacity to adsorb metal ions during soil interactions, thereby expanding their relevance for potential environmental remediation. The novelty of this study lies in its dual use of biowaste sources and in demonstrating their functional viability in soil conditions without the use of commercial polymer additives. The use of fruit peel polymers highlights the potential to contribute to circular economy approaches by valorizing waste. This study is positioned as a baseline material development and functional validation study, with detailed molecular characterization, adsorption quantification, and field-scale validation identified as key future research directions. Future research should explore field-scale deployment, incorporation of bioactive or nutrient compounds, recovery of adsorbed metal ions, and long-term performance metrics. These directions will help assess scalability and deepen the understanding of their role in sustainable agriculture, water conservation, and environmental detoxification.

## Supporting information

S1 Table Literature comparison of fruit waste-derived hydrogel and its relevance. (PDF)

S2 Table Raw experimental data used for statistical analysis. (XLSX)
