# Supplementary material for: Sustainable hydrogel from biowaste: Synthesis and characterization of pectin and starch-based hydrogels derived from fruit peel
Source: PLoS One. 2026 Jul 21;21(7):e0354244. doi: 10.1371/journal.pone.0354244 (PMC13387563; doi:10.1371/journal.pone.0354244)
Supplement: S1 Table — (PDF) [file pone.0354244.s001.pdf]

S1-Table. Literature comparison of fruit waste-derived hydrogel and its relevance.

Author	Material	Key Property	Application	Notes
**Error! Reference source not found.**Sulianto A. 2023	Pectin- starch hydrogel from fruit waste	Swelling Water retention Biodegradation	Retention of herbicide in soil	Composite shows better values in all parameters but no regional focus
**Error! Reference source not found.**Yusof et al. 2025	Pectin + starch DES	Swelling up to 108- 120%	Soil water retention	DES improves swelling and retention in soil simulation tests.
**Error! Reference source not found.**El-Aziz. 2022	Pectin- starch ecohydrogel	Water retention enhancement	Plant drought studies.	Includes plant test; no sorption studies.
**Error! Reference source not found.**Maia et al. 2024	Starch +banana peel activated carbon hydrogel	High heavy metal adsorption (Cr^{6+} upto 98%)	Water purification.	Combines starch + banana waste ; strong adsorption focus.
[84]Singha NR. 2017	Pectin -g- (sodium acrylate co-N-isopropylacrilamide) hydrogel.	Swelling studies- optimization of temperature and characteriation.	Superadsorption of dyes/M (II) ions	Emphazises the incorporation of natural polymer imparts a balance in adsorption efficiency
[85]Chauhan GS. 2007	Pectin and acrylamide based hydrogels.	High metal ion sorption (Pb^{2+}, Cd $^{2+}$)	Environmental remediation	Focus on adsorption; no soil or agricultural relevance.
[86]He et al. 2024	Mandarin peel composite hydrogel	Water retention enhancement	Plant drought studies	Include splant test no adsorption focus.

[87] Parlayıcı Ş, Baran Y. 2025	Waste banana peel/ Nano- Fe_3O_4/ alginate hydrogel beads.	High adsorption capacityfor Cr (VI)	Waste water treatment (heavy metal removal)	Magnetic hydrogel system enableseasy recovery; combines banana peel biosorption with alginate matrix, focused on adsorption, no soil water retention.
[88] Yue X. et al. 2018	Banana peel aerogel	High absorption capacity	Oil/water separation.	Shows that porous structure enhabces absorption.

DES- Deep Eutectic Solvent

Supplementary references.

[81] Merino D, Mansilla AY, Salcedo MF, Athanassiou A. Upcycling orange peel agricultural waste for the preparation of green hydrogels as active soil conditioners. ACS Sustain Chem Eng. 2023;11(29):10917–10928. https://doi.org/10.1021/acssuschemeng.3c02992

[82] Slezak R, Krzystek L, Puchalski M, Krucińska I, Sitarski A. Degradation of bio-based film plastics in soil under natural conditions. Sci Total Environ. 2023;866:161401. https://doi.org/10.1016/j.scitotenv.2023.161401

[83] Paswan M, Patel S, Prajapati V, Dholakiya BZ. Preparation and characterization of slow-release fertilizers loaded guar gum-g-poly methylmethacrylate-cl-polylactic acid (Gg-g-PMMA-cl-PLA) hydrogel and its effect on wheat growth. Int J Biol Macromol. 2023;253:126979. https://doi.org/10.1016/j.ijbiomac.2023.126979

[84] Singha NR, Karmakar M, Mahapatra M, Mondal H, Dutta A, Roy C, Chattopadhyay PK. Systematic synthesis of pectin-g-(sodium acrylate-co-N-isopropylacrylamide)

interpenetrating polymer network for superadsorption of dyes/M (II): determination of physicochemical changes in loaded hydrogels. Poly Chem. 2017;8(20):3211-37. https://doi.org/10.1039/C7PY00316A

[85] Chauhan GS, Kumari A, Sharma R. Pectin and acrylamide based hydrogels for environment management technologies: Synthesis, characterization, and metal ions sorption. Journal of applied polymer science. 2007;106(4):2158-68. https://doi.org/10.1002/app.26729

[86] He C, Wang X, Mou H, Hou W, He Q, Kang Y, Kong H, Li R, Chen W, Ao T, Li S. Sustainable soil rehabilitation with multifunctional mandarin orange peel/tobermorite composite hydrogels: Water retention, immobilization of heavy metals, fertilizer release and bacterial community composition. Chem Eng J. 2024;502:158030. https://doi.org/10.1016/j.cej.2024.158030

[87] Parlayıcı Ş, Baran Y. Removal of hexavalent chromium from aqueous solutions using nano-Fe3O4/waste banana peel/alginate hydrogel biobeads as adsorbent. Biomass Conversion and Biorefinery. 2025;15(12):18695-721. https://doi.org/10.1007/s13399-025-06489-6

[88] Yue X, Zhang T, Yang D, Qiu F, Li Z. Hybrid aerogels derived from banana peel and waste paper for efficient oil absorption and emulsion separation. Journal of Cleaner Production. 2018 Oct 20;199:411-9. https://doi.org/10.1016/j.jclepro.2018.07.181
