# Supplementary material for: Sustainable hydrogel from biowaste: Synthesis and characterization of pectin and starch-based hydrogels derived from fruit peel
Source: PLoS One. 2026 Jul 21;21(7):e0354244. doi: 10.1371/journal.pone.0354244 (PMC13387563; doi:10.1371/journal.pone.0354244)
Supplement: Attachment — Submitted filename: Reviewer_Comments_PONE-D-26-00016.docx [file pone.0354244.s003.docx]

COMMENTS FOR MANUSCRIPT PONE-D-26-00016

Sustainable Hydrogel from Biowaste: Synthesis and Characterization of Pectin and

Starch-Based Hydrogels Derived from Fruit Peel

Major Revision-

1. Similar pectin/starch hydrogels from fruit waste for swelling and soil retention already exist in literature. A) <https://doi.org/10.24191/srj.v22i1.17308> Preparation and Characterization of Pectin-Starch Hydrogel Modified with Urea and Lactic Acid-Based Deep Eutectic Solvent for Soil Water Retention

b) <https://doi.org/10.3390/cleantechnol6010001-> From Fruit Waste to Hydrogels for Agricultural Applications

Emphasize Gulf region novelty and highlight heavy metal adsorption as a unique contribution. Add Introduction. Reframe the discussion as the “first comprehensive Gulf study” and include a comparison table against the literature, which has a 65-75% topical overlap. Strengthen regional specificity for arid agriculture

2. Extraction and synthesis were performed under fixed conditions without statistical optimization (e.g., RSM or factorial design). Please justify parameter selection (e.g., preliminary trials or literature basis). Consider framing this as a baseline study and note in the discussion that optimization is recommended for future scale-up.

### 3. While pot experiments demonstrate improved water retention, plant growth indicators (e.g., germination, biomass, yield) are not included, limiting support for agricultural performance claims. The authors have **already acknowledged this limitation** in the discussion by noting controlled conditions, single soil type, and the need for broader climatic evaluation. This acknowledgement is appropriate. However, no additional experiments are required but ensure that claims throughout the manuscript remain **appropriately moderated** and consistent with the stated limitations.

### 4. Swelling behaviour was evaluated only in distilled water, which reduces applicability to saline or arid soils. Indicate this more explicitly as a limitation and recommend future testing under **ionic or saline conditions** representative of arid soils.

### 5. Consider including inferential statistics (e.g., ANOVA or t-tests) where applicable to support comparisons between hydrogel formulations and treatments.

## Minor Revisions

## Methods

- Specify **banana ripeness stage**, as starch yield is maturity-dependent in Lines 100-101.
- Provide **complete soil characterization** (pH, texture, moisture-holding capacity) in Lines 172–177.
- Recheck Lines 118–189 for clarity and consistency.

## Results

- Verify the reference in Line 222.
- Clarify what **250 min** represents (equilibrium time or t₉₀)?
- Include statistical significance for water-retention comparisons.
- Rephrase lines 299 and 320 for clarity.

Writing and Presentation

- The wording in Lines 93-98 is slightly inconsistent. Line 93 frames the work as a hypothesis, whereas Line 95 states that the study reports outcomes, which reads as conclusive. For clarity and a more conventional scientific tone, consider using a consistent objective-driven phrasing.
- Consider revising the title to reflect agricultural application. The paper states that hydrogels are made for agriculture, but the title doesn’t mention this.
- Units missing, for example, “2.16 pectin" → "2.16% pectin.”
- The pectin extraction was performed at a single fixed condition (pH 1.5, 90 °C) without optimization or justification; please provide a rationale or clarify that this represents baseline rather than optimized parameters like OFAT/OVAT as mentioned above in point 1.
- Since validation only utilized commercial potting soil, claims of UAE or Gulf desert applicability are not completely justified; consider modifying the claim or admitting the need for testing on representative soils (such as saline or sandy).
- Only post-burial EDX detection presently supports the adsorption claim; quantitative adsorption studies (capacity, kinetics, isotherms) or a toned-down claim is advised.
- Correct Figure 1 reference error (line 118)

Overall recommendation

Accept after moderate revisions

The study presents solid experimental work and contributes to sustainable hydrogel development from biowaste. However, clearer positioning relative to existing literature, improved methodological justification, moderated regional claims, and minor analytical clarifications are needed to strengthen the manuscript prior to publication.
