# Supplementary material for: Sustainable hydrogel from biowaste: Synthesis and characterization of pectin and starch-based hydrogels derived from fruit peel
Source: PLoS One. 2026 Jul 21;21(7):e0354244. doi: 10.1371/journal.pone.0354244 (PMC13387563; doi:10.1371/journal.pone.0354244)
Supplement: Attachment — Submitted filename: Response to Reviewers.docx [file pone.0354244.s005.docx]

**Response to the academic editor and reviewers’ comments**

**Manuscript Title**: Sustainable hydrogel from Biowaste: Synthesis and characterization of pectin and starch – based hydrogels derived from fruit peels.

We sincerely thank the Academic Editor and Reviewers for their constructive and insightful comments. We have carefully revised the manuscript to address all concerns. All changes have been incorporated and highlighted in the revised version. A detailed point-by-point response is provided below.

| **Academic Editor's additional requirements/comments** | **Response** |
| --- | --- |
| 1. Please ensure that your manuscript meets PLOS ONE's style requirements, including those for file naming. | The manuscript has been carefully revised to fully comply with PLOS ONE formatting and style guidelines, including file naming, figure formatting, and reference style. |
| 2. In your Methods section, please provide additional information regarding the permits you obtained for the work. Please ensure you have included the full name of the authority that approved the field site access and, if no permits were required, a brief statement explaining why. | No specific permits were required for this study, as all materials (fruit peels) were obtained from publicly accessible sources (local cafeterias and markets). A statement clarifying this has now been included in the Methods section. |
| 3. Thank you for stating the following financial disclosure:  “This work was financially supported by the Zayed University Research Incentive Fund (RIF) project-23075 awarded to M.A.Khan.”  Please state what role the funders took in the study. If the funders had no role, please state: "The funders had no role in study design, data collection and analysis, decision to publish, or preparation of the manuscript."  If this statement is not correct you must amend it as needed.  Please include this amended Role of Funder statement in your cover letter; we will change the online submission form on your behalf. | The funding statement has been revised to clearly specify the funder's role.  The Zayed University Research Incentive Fund (RIF project-23075) provided financial support for research personnel, consumables, minor equipment, and publication-related expenses. (This part probably can be relocated to the acknowledgment section.  The following statement has now been included:  “The funders had no role in study design, data collection and analysis, decision to publish, or preparation of the manuscript.”  This statement has also been incorporated into the cover letter, as requested. |
| 4. Please note that funding information should not appear in any section or other areas of your manuscript. We will only publish funding information present in the Funding Statement section of the online submission form. Please remove any funding-related text from the manuscript. | All funding-related text has been removed from the body of the manuscript and retained only in the Funding Statement section. |
| 5. We note that your Data Availability Statement is currently as follows:  “All relevant data are within the manuscript and its Supporting Information files.”  Please confirm at this time whether or not your submission contains all raw data required to replicate the results of your study. Authors must share the “minimal data set” for their submission. PLOS defines the minimal data set to consist of the data required to replicate all study findings reported in the article, as well as related metadata and methods (https://journals.plos.org/plosone/s/data-availability#loc-minimal-data-set-definition).  For example, authors should submit the following data:  - The values behind the means, standard deviations and other measures reported;  - The values used to build graphs;  - The points extracted from images for analysis.  Authors do not need to submit their entire data set if only a portion of the data was used in the reported study.  If your submission does not contain these data, please either upload them as Supporting Information files or deposit them to a stable, public repository and provide us with the relevant URLs, DOIs, or accession numbers. For a list of recommended repositories, please see https://journals.plos.org/plosone/s/recommended-repositories.  If there are ethical or legal restrictions on sharing a de-identified data set, please explain them in detail (e.g., data contain potentially sensitive information, data are owned by a third-party organization, etc.) and who has imposed them (e.g., an ethics committee). Please also provide contact information for a data access committee, ethics committee, or other institutional body to which data requests may be sent. If data are owned by a third party, please indicate how others may request data access. | All raw data supporting the findings (including values used for graphs, mean, and standard deviation calculations) have now been included as Supporting Information files. The Data Availability Statement has been updated accordingly to ensure compliance with PLOS ONE requirements. |
| 6. We note that Figures 1 and 5 in your submission contain copyrighted images. All PLOS content is published under the Creative Commons Attribution License (CC BY 4.0), which means that the manuscript, images, and Supporting Information files will be freely available online, and any third party is permitted to access, download, copy, distribute, and use these materials in any way, even commercially, with proper attribution. For more information, see our copyright guidelines: http://journals.plos.org/plosone/s/licenses-and-copyright.  We require you to either (1) present written permission from the copyright holder to publish these figures specifically under the CC BY 4.0 license, or (2) remove the figures from your submission:  1. You may seek permission from the original copyright holder of Figures 1 and 5 to publish the content specifically under the CC BY 4.0 license.  We recommend that you contact the original copyright holder with the Content Permission Form (http://journals.plos.org/plosone/s/file?id=7c09/content-permission-form.pdf) and the following text:  “I request permission for the open-access journal PLOS ONE to publish XXX under the Creative Commons Attribution License (CCAL) CC BY 4.0 (http://creativecommons.org/licenses/by/4.0/). Please be aware that this license allows unrestricted use and distribution, even commercially, by third parties. Please reply and provide explicit written permission to publish XXX under a CC BY license and complete the attached form.”  Please upload the completed Content Permission Form or other proof of granted permissions as an "Other" file with your submission.  In the figure caption of the copyrighted figure, please include the following text: “Reprinted from [ref] under a CC BY license, with permission from [name of publisher], original copyright [original copyright year].”  2. If you are unable to obtain permission from the original copyright holder to publish these figures under the CC BY 4.0 license or if the copyright holder’s requirements are incompatible with the CC BY 4.0 license, please either i) remove the figure or ii) supply a replacement figure that complies with the CC BY 4.0 license. Please check copyright information on all replacement figures and update the figure caption with source information. If applicable, please specify in the figure caption text when a figure is similar but not identical to the original image and is therefore for illustrative purposes only. | Figure 1 has been removed from the manuscript to ensure compliance with CC BY 4.0 requirements. Figure 5 has been carefully reviewed and confirmed to be an original figure created by the authors and does not contain any copyrighted material. Therefore, no permission is required.  All figures included in the revised manuscript are original and comply with PLOS ONE’s licensing requirements. |
| 7. If tables are embedded in the manuscript and ALSO loaded as separate files, please delete the separate files, leaving only the tables in the manuscript file | All tables have been reviewed, and any duplicate table files previously uploaded as separate files have been removed. The tables are now presented only within the manuscript, in accordance with PLOS ONE submission guidelines. |
| 8. If the reviewer comments include a recommendation to cite specific previously published works, please review and evaluate these publications to determine whether they are relevant and should be cited. There is no requirement to cite these works unless the editor has indicated otherwise. | All reviewer-suggested references have been carefully evaluated for relevance. Relevant and appropriate references have been incorporated into the revised manuscript where they strengthen the scientific context. References that were not directly relevant to the scope and objectives of this study were not included, in accordance with the editor’s guidance. |
| **Response to Peer reviewers’ comments** | |
| **Reviewer # 1 comment** | **Response** |
| 1. Page 3-4, Lines 52-88:The manuscript presents hydrogel synthesis using pectin and starch with calcium chloride and polyvinyl alcohol (PVA) crosslinking, which are well-established approaches. While the authors claim novelty in using "actual agro-waste," the technical innovation in the synthesis procedure itself is minimal. The introduction acknowledges that "most studies rely on commercially purified biopolymers," but the fundamental chemistry of hydrogel formation remains unchanged. | We agree that the fundamental chemistry of hydrogel formation is well established. Thus, the manuscript has been revised to clarify that the novelty of this work lies not in the synthesis chemistry, but in the agro-waste-derived pectin and starch, and in the evaluation of their functional and biodegradable properties in a sustainability-focused context. (Line 82- 87) |
| 2. Page 5-6, Lines 144-172: While the manuscript reports moisture content, ash content, and methoxyl content of pectin, the characterization is incomplete. Critical parameters such as degree of esterification (DE), molecular weight distribution, and degree of polymerization are not reported. For starch, the amylose-to-amylopectin ratio, gelatinization temperature, and crystallinity are absent. These parameters directly influence hydrogel properties and are essential for reproducibility. | We agree that additional characterization of pectin (degree of esterification, molecular weight distribution, and degree of polymerization) and starch (amylose:amylopectin ratio, gelatinization temperature, and crystallinity) would provide deeper insight into their structures and properties. In the present study, the primary focus was on evaluating the functional performances of the developed hydrogel under controlled conditions, including swelling behavior, water retention, and biodegradation, rather than molecular-level characterization of the raw material. We have included the pectin DE (Lines 363-367). Although comprehensive starch characterization was not conducted, the extraction method and source are consistent with those reported in previous studies that used banana peel starch to prepare hydrogels. The reproducibility of the hydrogel was ensured through controlled experimental conditions. We acknowledge this limitation and have added this in the limitation section in the revised manuscript (Lines 577-579). |
| 3. Page 6-7, Lines 140-143: The manuscript briefly mentions that "a few drops of 2 M NaOH" are added to induce gelation, but provides no mechanistic explanation. The crosslinking process is critical for determining hydrogel properties, yet the authors do not explain how calcium ions form ionic bridges or how pH adjustment facilitates gelation. The stoichiometry of the crosslinking reaction is not provided | Clarifying the gelation mechanism: In the present study, polyvinyl alcohol (PVA) was used as the primary crosslinking agent, forming a hydrogen-bonded, physically entangled network with starch to create a three-dimensional structure. A few drops of 2M NaOH were added to induce gelation by possibly adjusting the pH rather than acting as a crosslinking agent, similar to the mechanism reported in the mentioned literature (https://doi.org/10.1016/j.foodhyd.2019.05.046; already added in the manuscript ref [17]). (Line 170) |
| 4. Page 7-8, Lines 166-171: The swelling ratio measurement uses a filtration method with a tea bag, which is unconventional and raises concerns about accuracy. The manuscript does not explain how "blotting" is standardized; the duration, pressure, and material used for blotting are not specified. This lack of standardization compromises reproducibility and introduces significant experimental | The swelling ratio measurement has now been clarified to ensure reproducibility and standardization. Although the tea bag method was used as a simple filtration approach, the procedure was carefully controlled. Specifically, blotting was standardized by gently removing excess water from the surface under consistent conditions to avoid deformation of the hydrogel structure. The tea bag has been previously employed in hydrogel swelling studies as a practical means of separating free water without disrupting the gel network. To improve clarity and reproducibility, the detailed blotting conditions have now been explicitly described in the revised manuscript. (Lines 211-213) |
| 5. Page 8-9, Lines 173-178: While the manuscript mentions that "all experiments were performed in triplicate" and figures include error bars representing "mean standard error," no statistical tests (ANOVA, t-tests) are reported. The manuscript does not indicate whether differences between pectin and starch hydrogels are statistically significant. Additionally, the exact calculation method for standard error is not specified (standard deviation vs. standard error of the mean) | The manuscript has been revised to include appropriate statistical analysis (Line 262- 265). All results were performed in triplicate, and the results are expressed as mean± standard deviation. Statistical significance between pectin and starch hydrogels was evaluated using two- way ANOVA, followed by the Tukey Honest Significant Difference (HSD) test. Differences are considered statistically significant at p < 0.05.  The error bars presented in the figures represents standard deviation. |
| 6. Page 9, Lines 179-183: The biodegradation test buries hydrogels in soil for only 20 days at room temperature. This timeframe is insufficient to assess true biodegradability, especially for polysaccharide-based materials that may take months to degrade fully. Additionally, only one soil type and temperature condition are tested, limiting generalizability to the diverse environmental conditions mentioned in the introduction (arid regions with temperature fluctuations). | We agree that the 20-day biodegradation study is a short- term assessment and does not cover the extended degradation behavior. This experiment has been clearly repositioned as a baseline study intended to provide an initial indication of degradation behavior. The scope as well as the limitations of the study- mainly, short duration and controlled conditions have been acknowledged in the Results and Discussion section (Line 420- 423). We moderated the claims accordingly and clarified that a comprehensive evaluation under different environmental conditions (soil types, temperatures matching UAE weather, and long durations) is necessary for future work to fully standardize and assess environmental applicability, particularly in arid regions. (Line 544- 546) |
| 7. Page 15, Lines 300-313: The manuscript reports the emergence of elements (Fe, Cr, Cu, Zn) in EDX spectra after soil burial and claims "adsorption of heavy metals from the surrounding soil environment." However, this interpretation is speculative and lacks rigorous evidence to support it. The authors do not conduct controlled experiments with known heavy metal concentrations, nor do they quantify the amount of metals adsorbed. The EDX data alone cannot definitively prove adsorption versus mere surface contamination. | We agree that the EDX mapping alone cannot conclusively distinguish between true absorption and surface deposition, nor can it provide quantitative information on metal uptake. On this basis, the interpretation has been moderated accordingly in the revised manuscript. The presence of elements such as Fe, Cr, Cu, and Zn in the EDX spectra after soil burial is now considered indicative of possible interaction with soil-derived metal ions. To support this, an EDX spectrum comparison before and after soil burial has been included, clearly showing their presence after soil interaction. Similar approaches have been reported in the literature, where EDX is used as a primary tool to indicate surface association of metal ions in hydrogel – soil interaction studies. Even though adsorption studies require controlled experiments and quantitative analysis. (Line 451- 484). This point is also mentioned as a limitation (Line 552- 555) |
| 8. Page 16, Lines 338-343: The manuscript describes visual changes after soil burial (pectin turning from orange to pale yellow, starch turning black) but does not systematically compare the structural integrity or functional performance of the two hydrogels. The statement that starch hydrogels "largely retain their structural form" while pectin hydrogels show "cross-link loss" is not quantitatively supported. This comparison is crucial for determining which hydrogel is more suitable for agricultural applications. | We agree that visual changes alone are insufficient to support conclusions about structural integrity and functional use. In the revised manuscript, the interpretation has been refined to depend on quantitative biodegradation percentage data analysed using ANOVA rather than qualitative observation alone. The result shows that both time and hydrogel type significantly affect degradation, with starch hydrogel exhibiting higher degradation rates. The previous statements regarding structural integrity have been revised to reflect these observations. (Line 529-536) |
| 9. Page 17, Lines 358-377: While the conclusion mentions "practical applications" and "soil moisture retention," the manuscript provides limited evidence that these hydrogels would function effectively in real agricultural settings. The water retention experiment uses only 0.34 g of hydrogel mixed with 170 g of soil; a ratio that may not reflect practical field application rates. The manuscript does not discuss optimal hydrogel loading rates, application methods, or long-term performance in actual crops. | In our study, the hydrogel-to-soil ratio (0.34g per 170 g soil) matches approximately 2g per kg soil, which falls within the range reported in the literature for agricultural applications (https://doi.org/10.1016/j.scienta.2024.113305). The application range of 1-3 g/kg of soil has been reported to improve soil water retention as well as plant growth. The selected dosage is consistent with reported application ranges and can be considered representative of realistic use, given that our study is at the laboratory scale. We acknowledge that factors such as field study under varying salinity and temperature, and long-term crop performance, require further investigation. These limitations have now been stated in the revised manuscript. (Line 542- 564). |
| 10. Page 3-4, Lines 52-70: The manuscript extensively discusses the benefits of biodegradable hydrogels but does not compare the performance of the synthesized pectin and starch hydrogels with commercially available hydrogels (both synthetic and natural). Without such comparison, it is difficult to assess whether the fruit peel-derived hydrogels offer genuine advantages beyond waste valorization. | We agree that commercial hydrogel is necessary to interpret material performance. In this study, the primary objective was to develop biodegradable hydrogels from fruit peel-derived biopolymer and evaluate their functional properties. As a direct experimental comparison with a synthetic hydrogel was not conducted, relevant literature comparisons have now been included in the revised manuscript. (Lines 448-453). Even though commercial hydrogels exhibit higher mechanical strength and swelling capacity, this can reduce biodegradability and raise environmental concerns. In contrast, the developed hydrogel demonstrates good swelling, water retention, and biodegradability, highlighting its benefit as a sustainable alternative. |
| 11. Page 17, Lines 371-377: The manuscript claims alignment with "circular economy models" and "green policy development," but provides no quantitative assessment of environmental benefits. The authors do not calculate the carbon footprint, water usage, or waste reduction achieved by using fruit peel waste compared to conventional hydrogel production. The energy requirements for extraction, synthesis, and processing are not discussed. | We acknowledge that quantitative assessments of environmental impacts, such as carbon footprint, water use, and energy requirements, will provide a comprehensive evaluation of sustainability. In the present study, the focus was on the development and functional characterization of biodegradable hydrogels. The statement mentioning circular economy and green policy has been revised to reflect potential environmental benefits rather than claims. (Line 610- 611) |
| 12- Page 15-16, Lines 318-336: The FTIR analysis describes peak positions and assignments for both hydrogels but lacks depth in interpreting the significance of observed changes after biodegradation. The statement that "postburial FTIR spectra revealed shifts and reductions in peak intensities" is vague and lacks quantitative analysis. The authors do not explain which functional groups are preferentially degraded or why, nor do they correlate FTIR changes with weight loss data. | We acknowledge that a deeper interpretation of FTIR results is important for a better understanding of hydrogel biodegradation. On this basis, in the revised manuscript, we have expanded the FTIR discussion. In particular, we have interpreted the reduction in hydroxyl and carboxyl group intensities as evidence of preferential degradation of hydrophilic groups; meanwhile, the change in glycosidic bond peaks indicates polysaccharide backbone cleavage. (Line 514, pages 20 and 21) |
| 13. Page 14-15, Lines 285-296: The SEM images are presented with qualitative descriptions (e.g., "highly interconnected porous morphology") but lack quantitative analysis. Pore size distribution, porosity percentage, and surface area are not reported. These parameters are critical for understanding swelling behavior and degradation kinetics, yet they are not measured or discussed | We agree that quantitative analysis of pore size distribution, porosity, and surface area would provide deeper insight into the hydrogel's structure and its role in swelling, degradation, and water retention. In the present work, SEM analysis was primarily used for qualitative assessment of surface morphology. In the revised manuscript, this has now been acknowledged as a limitation, and future work will focus on incorporating detailed quantification to correlate structure and functional relationship. (Line 587-587) |
| 14. Page 8-9, Lines 172-178: The manuscript reports water retention percentages but does not explain the underlying mechanisms. The authors do not discuss whether water is retained through capillary forces, hydrogen bonding, or osmotic effects. The relationship between hydrogel swelling capacity and water retention in soil is assumed but not experimentally validated | We agree that a clear explanation of the mechanism governing water retention is important. In the revised manuscript, we included a discussion of the roles of hydrogen bonding, osmotic pressure, and capillary forces in water retention and swelling. The contribution of hydrophilic groups (-OH and -COO), evident through FTIR, has been discussed in relation to swelling, and the porous morphology observed in SEM has been linked to capillary water retention. Relevant literature has been incorporated to support these mechanisms. While direct experimental validation of each mechanism was beyond the scope of this study, the discussion has been revised to give a literature-supported interpretation. (Line 553-563) |
| 15. Page 16, Lines 347-355: While the manuscript acknowledges some limitations (controlled laboratory conditions, single soil type), the discussion is superficial. The authors mention exploring "mango, papaya, watermelon" as alternative fruit sources but do not discuss other critical limitations such as seasonal variability in fruit peel composition, scaling challenges, or regulatory considerations for agricultural use. | We acknowledge that more practical and real-world limitations should be discussed to better frame the applicability of the developed hydrogel. In the revised manuscript, the limitations section has been expanded to include variation in fruit peel composition, seasonal availability, and potential for large-scale production. These points are now briefly addressed to provide a more comprehensive perspective of the future development of the hydrogel for agricultural applications. (Line 603-613) |
| 16. Page 6-7, Lines 140-143: The manuscript does not characterize the crosslink density, network structure, or gel fraction of the synthesized hydrogels. These parameters are fundamental to understanding hydrogel properties and are typically reported for hydrogel studies. Without this information, it is impossible to assess whether the crosslinking was successful or to predict hydrogel behavior. | The major parameters, such as crosslink density, network structure, and gel fraction, are very important for the detailed characterization of the developed hydrogel. However, the primary focus of the present study was on evaluating the performance of the developed hydrogel, including swelling percentage, water retention, and biodegradability. Direct characterization of crosslink density and gel fraction was not performed, as this is a baseline/preliminary study. This has been acknowledged as a limitation, and future work will aim to add further detailed characterizations. (Line 615- 616) |
| 17. Page 17, Lines 371-377: The manuscript proposes using fruit peel-derived hydrogels in agricultural systems but does not address regulatory approval, food safety, or potential toxicity concerns. If these hydrogels are used in food crop production, regulatory agencies may require safety assessments. The presence of residual extraction chemicals or microbial contaminants is not discussed | We recognize that factors such as food safety, regulatory approval, and toxicity are major considerations for agricultural applications. As the present study does not involve actual agricultural applications, these considerations were not evaluated at the laboratory scale. Points such as residual chemicals from the hydrogel, microbial contamination, and recovery of adsorbed metal ions from the gel need to be addressed. In the manuscript, these points have been acknowledged in the limitations section. (Line 605-607) |
| 18. Page 8, Lines 166-171: The manuscript reports final swelling ratios but does not analyze swelling kinetics. The time-dependent swelling data are presented in Figure 2 but not discussed in detail. The authors do not report swelling half-times, maximum swelling rates, or whether swelling follows predictable kinetic models (e.g., Fickian or anomalous diffusion). | We agree that the swelling kinetics analysis, including swelling half-time, maximum swelling rate, and diffusion modeling, would provide a deeper understanding of the swelling mechanism. In this study, the focus was on equilibrium swelling behavior and on comparing hydrogel performance rather than on kinetic modeling of swelling. Although time- dependent swelling data were presented, deeper kinetic modeling was not performed. This has now been acknowledged in the revised manuscript as a limitation. (Line 559- 560) |
| 19. Page 14-16, Lines 285-336: The manuscript presents extensive characterization data (SEM, EDX, FTIR) but fails to establish clear correlations between structural properties and functional performance (swelling, water retention, degradation). For example, the relationship between pore size (from SEM) and swelling capacity is not discussed. The role of functional groups (from FTIR) in water absorption is not analyzed | We agree that correlating structural characteristics with functional performance will strengthen the interpretation of results. In the revised manuscript, we have expanded the discussion part to connect the FTIR, SEM, and functional properties. Specifically, the FTIR was used to relate the presence of hydrophilic groups (-OH and -COOH-) to water absorption and swelling behavior, while the porous structure observed with SEM was linked to water uptake and retention via capillary effects. These features have been discussed in the literature with respect to swelling, water retention, and biodegradation to provide a more integrated interpretation of the results. (Line 514- 539) |
| 20. Page 3-4, Lines 52-88: The manuscript does not reference or discuss recent advances in sustainable hydrogel materials and molecular reconstruction techniques. Recent publications on advanced hydrogel synthesis and property enhancement are not cited, limiting the contextual positioning of this work within the broader field | We agree that positioning the study within recent advances is important. The Introduction has been revised to include recent literature on sustainable hydrogel development, including advances in bio-based hydrogels, composite systems, and waste-derived materials. However, since the primary focus of this study is resource valorization rather than advanced molecular reconstruction, the discussion has been kept aligned with the study objectives. In the revised manuscript, we have added recent literature to interpret and discuss the results section.   1) Parlayıcı Ş, Baran Y. Fruit peel incorporated alginate based magnetic hydrogel bio-composite beads for removal of hexavalent chromium. International Journal of Biological Macromolecules. 2025;284:137946. <https://doi.org/10.1016/j.ijbiomac.2024.137946>, 2) Zayed AM, Hussein AA, Sabra SA. Alginate/pectin hydrogel film incorporating atorvastatin and Jacaranda mimosifolia leaf extract for wound healing: Preparation, characterization and in vivo evaluation. Int J Bio Mac mol. 2025;14:146895. <https://doi.org/10.1016/j.ijbiomac.2025.146895. 3>) 2) Takahara Y, Beni Y, Sekine Y, Nankawa T, Ikeda-Fukazawa T. Structural changes of water in carboxymethyl cellulose nanofiber hydrogels during vapor swelling and drying. ACS omega. 2024 Oct 29;9(45):45554-63. <https://doi.org/10.1021/acsomega.4c07831> |

| **Reviewer # 2 comment** | **Response** |
| --- | --- |
| 1. Similar studies exist, emphasize Gulf novelty, heavy metal adsorption, add a comparison table. | We agree that there is a substantial body of literature reporting pectin and starch based hydrogels derived from fruit waste for swelling and water retention applications, even though our work shares a thematic overlap with existing studies, the novelty of the present study lies in- In corporation of heavy metal adsorption and we consider this as a baseline study for future update to hydrogel for Gulf arid conditions and agricultural needs. For comparison, a supplementary table (Table 2) has been added to highlight both the overlapping and distinguishing features of this work. The claims have been moderated to reflect this as a study based on laboratory-scale evaluation. |
| 2. Extraction and synthesis were performed under fixed conditions without statistical optimization (e.g., RSM or factorial design). Please justify parameter selection (e.g., preliminary trials or literature basis). Consider framing this as a baseline study and note in the discussion that optimization is recommended for future scale-up. | In response, we have revised the manuscript to clarify the extraction conditions (pH and temperature). We chose this extraction parameter based on a previous literature review,  <https://doi.org/10.3390/app11146596>, which summarizes the commonly applied conditions for controlled extraction. Accordingly in the manuscript, Result and Discussion, we have clearly stated this work as a baseline study (Lines 256-262). |
| 3. While pot experiments demonstrate improved water retention, plant growth indicators (e.g., germination, biomass, yield) are not included, limiting support for agricultural performance claims. The authors have already acknowledged this limitation in the discussion by noting controlled conditions, single soil type, and the need for broader climatic evaluation. This acknowledgement is appropriate. However, no additional experiments are required but ensure that claims throughout the manuscript remain appropriately moderated and consistent with the stated limitations. | To address this constructive feedback, we have carefully revised the manuscript to ensure that all claims related to real-life application on agricultural performance are appropriately moderated and fully consistent with the scope of the present study. Specifically, statements implying direct impact on plant growth have been rephrased to clearly state that the material's performance is under controlled conditions throughout the Introduction, Results and Discussion, Conclusion, and Limitations sections. |
| 4. Swelling behaviour was evaluated only in distilled water, which reduces applicability to saline or arid soils. Indicate this more explicitly as a limitaion and recommend future testing under ionic and saline conitions representative of arid soil. | We agree that evaluating swelling behavior solely in distilled water is a limitation for arid land applicability. Accordingly, we have revised the manuscript to address this point. In the Discussion section (Lines 319-329), a paragraph has been added to explain that the swelling performance observed in distilled water may not fully reflect behavior under salinity-stress conditions. In addition, a statement has been added to the limitations section (Lines 492-495) to clearly acknowledge that swelling was assessed only under distilled water conditions. |
| 5. Consider including inferential statistics (e.g., ANOVA or t-tests) where applicable to support comparisons between hydrogel formulations and treatments. | Statistical analyses, including two-way ANOVA and Tukey post hoc tests, have now been included. Accordingly, the method and results section, including images, have been updated. |
| **Minor Revisions** | |
| **Methods** | |
| • Specify banana ripeness stage, as starch yield is maturity-dependent in Lines 100-101. | The ripeness stage has been added in the Methods section. (Line 106) |
| • Provide complete soil characterization (pH, texture, moisture-holding capacity) in Lines 172–177. | We have included the soil physicochemical properties in the revised manuscript (now in line 207-208) |
| • Recheck Lines 118–189 for clarity and consistency. | This section has been revised for clarity and consistency. |
| **Results** | |
| • Verify the reference in Line 222. | The reference has been corrected. |
| • Clarify what 250 min represents (equilibrium time or t₉₀)? | This has been clarified as equilibrium swelling time (line- 312). |
| • Include statistical significance for water-retention comparisons. | Statistical analysis has been included using ANOVA and Tukey post hoc in the results section as well as in the graphs. |
| • Rephrase lines 299 and 320 for clarity. | The rephrasing has been done to improve clarity. Line 299 became 387, and line 408 is line 452 after additions. |
| **Writing and Presentation** | |
| • The wording in Lines 93-98 is slightly inconsistent. Line 93 frames the work as a hypothesis, whereas Line 95 states that the study reports outcomes, which reads as conclusive. For clarity and a more conventional scientific tone, consider using a consistent objective-driven phrasing. | The introduction section has been revised to maintain an objective-driven tone. Fruit peels, including banana and citrus, account for 25-30% of fruit and vegetable processing waste. This highlights the need to use fruit waste to develop eco-friendly, value-added products. The present study focuses on utilizing the fruit waste, which is abundant in natural polymers that can be transformed into a hydrogel with beneficial properties for agricultural applications, such as soil moisture retention, as well as biodegradability, which reduces product retention in soil post-use. At this point, we did not focus on the field trial; our aim was to validate the feasibility of hydrogel production and assess the potential for utilizing waste materials. Detailed chemical characterization of the extracted material, as well as the pot experiments, is under consideration/ being investigated. (line 92-100). |
| • Consider revising the title to reflect agricultural application. The paper states that hydrogels are made for agriculture, but the title doesn’t mention this. | We thank the reviewer for this valuable suggestion. We agree that highlighting agricultural applications in the title could improve clarity. However, the present study is primarily focused on the synthesis and baseline characterization of hydrogels derived from fruit peel biowaste, with agricultural applications demonstrated under controlled laboratory conditions rather than field-scale or crop-based validation.  To avoid overstating the scope of the work, we have retained the original title. Nevertheless, we have revised the multiple sections of the manuscript to more clearly emphasize the potential agricultural relevance and applications of the developed hydrogels. |
| • Units missing, for example, “2.16 pectin" → "2.16% pectin.” | Missing units have been updated. |
| • The pectin extraction was performed at a single fixed condition (pH 1.5, 90 °C) without optimization or justification; please provide a rationale or clarify that this represents baseline rather than optimized parameters like OFAT/OVAT as mentioned above in point 1. | The pectin extraction was performed at a fixed condition (pH 1.5, 90 °C) based on literature-reported conditions. We have included the related reference in the Results and Discussion section. Here, the pectin was extracted at fixed conditions to serve as a baseline for hydrogel preparation. It has been previously reported that the extraction of pectin from grapefruit, citron, and pomelo peels resulted in higher yields when an aqueous solution with pH 1.5 was used, compared to extractions performed within a wider range of 1.5- 4.2. (line 258-264). |
| • Since validation only utilized commercial potting soil, claims of UAE or Gulf desert applicability are not completely justified; consider modifying the claim or admitting the need for testing on representative soils (such as saline or sandy). | We acknowledge that the current validations were conducted only using commercial potting soil. This limits the claims regarding the applicability of the developed hydrogel to UAE weather conditions. The manuscript has been modified to clarify this limitation and has suggested future studies. While the hydrogels exhibited promising biodegradation in potting mix, their degradation nature in saline soil and high-temperature weather may differ, which highlights the need for further testing under field conditions (lines 377-379). Along with this, a concise limitation statement has been. “In order to assess the adaptability of hydrogels in case of biodegradation to the real UAE weather and soil conditions, further evaluation is necessary on representative arid soil conditions (lines 499-501) |
| • Only post-burial EDX detection presently supports the adsorption claim; quantitative adsorption studies (capacity, kinetics, isotherms) or a toned-down claim is advised. | We agree that the EDX analysis provides only a qualitative evaluation; accordingly, we have revised the manuscript to moderate the adsorption-related claim. The result section has been updated to clearly state that EDX analysis indicated the presence of metal ions on the hydrogel after soil burial, suggesting possible adsorption or interaction with soil components (lines 406-407). EDX provides qualitative evidence but does not quantify adsorption capacity. So further studies involving adsorption kinetics and capacity determination are required to completely assess the performance of the developed hydrogel (line 432- 435) |
| • Correct Figure 1 reference error (line 118) | The figure reference has been corrected in the image file. |
| **Overall recommendation** |  |
| Accept after moderate revisions  The study presents solid experimental work and contributes to sustainable hydrogel development from biowaste. However, clearer positioning relative to existing literature, improved methodological justification, moderated regional claims, and minor analytical clarifications are needed to strengthen the manuscript prior to publication. | We believe these revisions have significantly improved our manuscript. |
