# Supplementary material for: Sustainable hydrogel from biowaste: Synthesis and characterization of pectin and starch-based hydrogels derived from fruit peel
Source: PLoS One. 2026 Jul 21;21(7):e0354244. doi: 10.1371/journal.pone.0354244 (PMC13387563; doi:10.1371/journal.pone.0354244)
Supplement: Peer review history

# Peer review: Decision Letter 0

**DOI:** 10.1371/journal.pone.0354244.r001

**Authors:** Debajyoti Kundu

12 Mar 2026

PONE-D-26-00016

Sustainable Hydrogel from Biowaste: Synthesis and Characterization of Pectin and Starch-Based Hydrogels Derived from Fruit Peel

PLOS One

Dear Dr. KHAN,

Thank you for submitting your manuscript to PLOS ONE. After careful consideration, we feel that it has merit but does not fully meet PLOS ONE’s publication criteria as it currently stands. Therefore, we invite you to submit a revised version of the manuscript that addresses the points raised during the review process.

==============================

**ACADEMIC EDITOR:** The manuscript addresses an interesting topic on biowaste-derived hydrogels; however, important methodological details, statistical validation, and deeper material characterization are lacking. Clarification of experimental design, stronger analysis of hydrogel properties, and improved discussion of agricultural applicability are required before the manuscript can be considered for publication.

==============================

Please submit your revised manuscript by Apr 26 2026 11:59PM. If you will need more time than this to complete your revisions, please reply to this message or contact the journal office at plosone@plos.org. When you're ready to submit your revision, log on to https://www.editorialmanager.com/pone/ and select the 'Submissions Needing Revision' folder to locate your manuscript file.

Please include the following items when submitting your revised manuscript:

- A letter that responds to each point raised by the academic editor and reviewer(s). You should upload this letter as a separate file labeled 'Response to Reviewers'.
- A marked-up copy of your manuscript that highlights changes made to the original version. You should upload this as a separate file labeled 'Revised Manuscript with Track Changes'.
- An unmarked version of your revised paper without tracked changes. You should upload this as a separate file labeled 'Manuscript'.

If you would like to make changes to your financial disclosure, please include your updated statement in your cover letter. Guidelines for resubmitting your figure files are available below the reviewer comments at the end of this letter.

If applicable, we recommend that you deposit your laboratory protocols in protocols.io to enhance the reproducibility of your results. Protocols.io assigns your protocol its own identifier (DOI) so that it can be cited independently in the future. For instructions see: https://journals.plos.org/plosone/s/submission-guidelines#loc-laboratory-protocols. Additionally, PLOS ONE offers an option for publishing peer-reviewed Lab Protocol articles, which describe protocols hosted on protocols.io. Read more information on sharing protocols at https://plos.org/protocols?utm_medium=editorial-email&utm_source=authorletters&utm_campaign=protocols.

We look forward to receiving your revised manuscript.

Kind regards,

Debajyoti Kundu, Ph.D

Academic Editor

PLOS One

**Journal Requirements:**

When submitting your revision, we need you to address these additional requirements.

1. Please ensure that your manuscript meets PLOS ONE's style requirements, including those for file naming. The PLOS ONE style templates can be found at

https://journals.plos.org/plosone/s/file?id=wjVg/PLOSOne_formatting_sample_main_body.pdf and

https://journals.plos.org/plosone/s/file?id=ba62/PLOSOne_formatting_sample_title_authors_affiliations.pdf

2. In your Methods section, please provide additional information regarding the permits you obtained for the work. Please ensure you have included the full name of the authority that approved the field site access and, if no permits were required, a brief statement explaining why.

3. Thank you for stating the following financial disclosure:

“This work was financially supported by the Zayed University Research Incentive Fund (RIF) project-23075 awarded to M.A.Khan.”

Please state what role the funders took in the study.  If the funders had no role, please state: "The funders had no role in study design, data collection and analysis, decision to publish, or preparation of the manuscript."

If this statement is not correct you must amend it as needed.

Please include this amended Role of Funder statement in your cover letter; we will change the online submission form on your behalf.

4. Please note that funding information should not appear in any section or other areas of your manuscript. We will only publish funding information present in the Funding Statement section of the online submission form. Please remove any funding-related text from the manuscript.

5. We note that your Data Availability Statement is currently as follows:

“All relevant data are within the manuscript and its Supporting Information files.”

Please confirm at this time whether or not your submission contains all raw data required to replicate the results of your study. Authors must share the “minimal data set” for their submission. PLOS defines the minimal data set to consist of the data required to replicate all study findings reported in the article, as well as related metadata and methods (https://journals.plos.org/plosone/s/data-availability#loc-minimal-data-set-definition).

For example, authors should submit the following data:

- The values behind the means, standard deviations and other measures reported;

- The values used to build graphs;

- The points extracted from images for analysis.

Authors do not need to submit their entire data set if only a portion of the data was used in the reported study.

If your submission does not contain these data, please either upload them as Supporting Information files or deposit them to a stable, public repository and provide us with the relevant URLs, DOIs, or accession numbers. For a list of recommended repositories, please see https://journals.plos.org/plosone/s/recommended-repositories.

If there are ethical or legal restrictions on sharing a de-identified data set, please explain them in detail (e.g., data contain potentially sensitive information, data are owned by a third-party organization, etc.) and who has imposed them (e.g., an ethics committee). Please also provide contact information for a data access committee, ethics committee, or other institutional body to which data requests may be sent. If data are owned by a third party, please indicate how others may request data access.

6. We note that Figures 1 and 5 in your submission contain copyrighted images. All PLOS content is published under the Creative Commons Attribution License (CC BY 4.0), which means that the manuscript, images, and Supporting Information files will be freely available online, and any third party is permitted to access, download, copy, distribute, and use these materials in any way, even commercially, with proper attribution. For more information, see our copyright guidelines: http://journals.plos.org/plosone/s/licenses-and-copyright.

We require you to either (1) present written permission from the copyright holder to publish these figures specifically under the CC BY 4.0 license, or (2) remove the figures from your submission:

1. You may seek permission from the original copyright holder of Figures 1 and 5 to publish the content specifically under the CC BY 4.0 license.

We recommend that you contact the original copyright holder with the Content Permission Form (http://journals.plos.org/plosone/s/file?id=7c09/content-permission-form.pdf) and the following text:

“I request permission for the open-access journal PLOS ONE to publish XXX under the Creative Commons Attribution License (CCAL) CC BY 4.0 (http://creativecommons.org/licenses/by/4.0/). Please be aware that this license allows unrestricted use and distribution, even commercially, by third parties. Please reply and provide explicit written permission to publish XXX under a CC BY license and complete the attached form.”

Please upload the completed Content Permission Form or other proof of granted permissions as an "Other" file with your submission.

In the figure caption of the copyrighted figure, please include the following text: “Reprinted from [ref] under a CC BY license, with permission from [name of publisher], original copyright [original copyright year].”

2. If you are unable to obtain permission from the original copyright holder to publish these figures under the CC BY 4.0 license or if the copyright holder’s requirements are incompatible with the CC BY 4.0 license, please either i) remove the figure or ii) supply a replacement figure that complies with the CC BY 4.0 license. Please check copyright information on all replacement figures and update the figure caption with source information. If applicable, please specify in the figure caption text when a figure is similar but not identical to the original image and is therefore for illustrative purposes only.

7. If tables are embedded in the manuscript and ALSO loaded as separate files, please delete the separate files, leaving only the tables in the manuscript file.

8. If the reviewer comments include a recommendation to cite specific previously published works, please review and evaluate these publications to determine whether they are relevant and should be cited. There is no requirement to cite these works unless the editor has indicated otherwise.

**Additional Editor Comments:**

The manuscript addresses an interesting topic on biowaste-derived hydrogels; however, important methodological details, statistical validation, and deeper material characterization are lacking. Clarification of experimental design, stronger analysis of hydrogel properties, and improved discussion of agricultural applicability are required.

[Note: HTML markup is below. Please do not edit.]

Reviewers' comments:

Reviewer's Responses to Questions

**Comments to the Author**

1. Is the manuscript technically sound, and do the data support the conclusions?

The manuscript must describe a technically sound piece of scientific research with data that supports the conclusions. Experiments must have been conducted rigorously, with appropriate controls, replication, and sample sizes. The conclusions must be drawn appropriately based on the data presented.

Reviewer #1: No

Reviewer #2: Yes

**********

2. Has the statistical analysis been performed appropriately and rigorously?

Reviewer #1: No

Reviewer #2: No

**********

3. Have the authors made all data underlying the findings in their manuscript fully available?

The PLOS Data policy requires authors to make all data underlying the findings described in their manuscript fully available without restriction, with rare exception (please refer to the Data Availability Statement in the manuscript PDF file). The data should be provided as part of the manuscript or its supporting information, or deposited to a public repository. For example, in addition to summary statistics, the data points behind means, medians and variance measures should be available. If there are restrictions on publicly sharing data—e.g. participant privacy or use of data from a third party—those must be specified.

Reviewer #1: No

Reviewer #2: Yes

**********

4. Is the manuscript presented in an intelligible fashion and written in standard English?

PLOS ONE does not copyedit accepted manuscripts, so the language in submitted articles must be clear, correct, and unambiguous. Any typographical or grammatical errors should be corrected at revision, so please note any specific errors here.

Reviewer #1: Yes

Reviewer #2: Yes

**********

5. Review Comments to the Author

Please use the space provided to explain your answers to the questions above. You may also include additional comments for the author, including concerns about dual publication, research ethics, or publication ethics. (Please upload your review as an attachment if it exceeds 20,000 characters)

Reviewer #1: 1- Page 3-4, Lines 52-88:The manuscript presents hydrogel synthesis using pectin and starch with calcium chloride and polyvinyl alcohol (PVA) crosslinking, which are well-established approaches. While the authors claim novelty in using "actual agro-waste," the technical innovation in the synthesis procedure itself is minimal. The introduction acknowledges that "most studies rely on commercially purified biopolymers," but the fundamental chemistry of hydrogel formation remains unchanged.

2- Page 5-6, Lines 144-172: While the manuscript reports moisture content, ash content, and methoxyl content of pectin, the characterization is incomplete. Critical parameters such as degree of esterification (DE), molecular weight distribution, and degree of polymerization are not reported. For starch, the amylose-to-amylopectin ratio, gelatinization temperature, and crystallinity are absent. These parameters directly influence hydrogel properties and are essential for reproducibility.

3- Page 6-7, Lines 140-143: The manuscript briefly mentions that "a few drops of 2 M NaOH" are added to induce gelation, but provides no mechanistic explanation. The crosslinking process is critical for determining hydrogel properties, yet the authors do not explain how calcium ions form ionic bridges or how pH adjustment facilitates gelation. The stoichiometry of the crosslinking reaction is not provided.

4- Page 7-8, Lines 166-171: The swelling ratio measurement uses a filtration method with a tea bag, which is unconventional and raises concerns about accuracy. The manuscript does not explain how "blotting" is standardized; the duration, pressure, and material used for blotting are not specified. This lack of standardization compromises reproducibility and introduces significant experimental error.

5- Page 8-9, Lines 173-178: While the manuscript mentions that "all experiments were performed in triplicate" and figures include error bars representing "mean standard error," no statistical tests (ANOVA, t-tests) are reported. The manuscript does not indicate whether differences between pectin and starch hydrogels are statistically significant. Additionally, the exact calculation method for standard error is not specified (standard deviation vs. standard error of the mean)

6- Page 9, Lines 179-183: The biodegradation test buries hydrogels in soil for only 20 days at room temperature. This timeframe is insufficient to assess true biodegradability, especially for polysaccharide-based materials that may take months to degrade fully. Additionally, only one soil type and temperature condition are tested, limiting generalizability to the diverse environmental conditions mentioned in the introduction (arid regions with temperature fluctuations).

7- Page 15, Lines 300-313: The manuscript reports the emergence of elements (Fe, Cr, Cu, Zn) in EDX spectra after soil burial and claims "adsorption of heavy metals from the surrounding soil environment." However, this interpretation is speculative and lacks rigorous evidence to support it. The authors do not conduct controlled experiments with known heavy metal concentrations, nor do they quantify the amount of metals adsorbed. The EDX data alone cannot definitively prove adsorption versus mere surface contamination.

8- Page 16, Lines 338-343: The manuscript describes visual changes after soil burial (pectin turning from orange to pale yellow, starch turning black) but does not systematically compare the structural integrity or functional performance of the two hydrogels. The statement that starch hydrogels "largely retain their structural form" while pectin hydrogels show "cross-link loss" is not quantitatively supported. This comparison is crucial for determining which hydrogel is more suitable for agricultural applications.

9- Page 17, Lines 358-377: While the conclusion mentions "practical applications" and "soil moisture retention," the manuscript provides limited evidence that these hydrogels would function effectively in real agricultural settings. The water retention experiment uses only 0.34 g of hydrogel mixed with 170 g of soil; a ratio that may not reflect practical field application rates. The manuscript does not discuss optimal hydrogel loading rates, application methods, or long-term performance in actual crops.

10- Page 3-4, Lines 52-70: The manuscript extensively discusses the benefits of biodegradable hydrogels but does not compare the performance of the synthesized pectin and starch hydrogels with commercially available hydrogels (both synthetic and natural). Without such comparison, it is difficult to assess whether the fruit peel-derived hydrogels offer genuine advantages beyond waste valorization.

11- Page 17, Lines 371-377: The manuscript claims alignment with "circular economy models" and "green policy development," but provides no quantitative assessment of environmental benefits. The authors do not calculate the carbon footprint, water usage, or waste reduction achieved by using fruit peel waste compared to conventional hydrogel production. The energy requirements for extraction, synthesis, and processing are not discussed.

12- Page 15-16, Lines 318-336: The FTIR analysis describes peak positions and assignments for both hydrogels but lacks depth in interpreting the significance of observed changes after biodegradation. The statement that "postburial FTIR spectra revealed shifts and reductions in peak intensities" is vague and lacks quantitative analysis. The authors do not explain which functional groups are preferentially degraded or why, nor do they correlate FTIR changes with weight loss data.

13- Page 14-15, Lines 285-296: The SEM images are presented with qualitative descriptions (e.g., "highly interconnected porous morphology") but lack quantitative analysis. Pore size distribution, porosity percentage, and surface area are not reported. These parameters are critical for understanding swelling behavior and degradation kinetics, yet they are not measured or discussed.

14- Page 8-9, Lines 172-178: The manuscript reports water retention percentages but does not explain the underlying mechanisms. The authors do not discuss whether water is retained through capillary forces, hydrogen bonding, or osmotic effects. The relationship between hydrogel swelling capacity and water retention in soil is assumed but not experimentally validated.

15- Page 16, Lines 347-355: While the manuscript acknowledges some limitations (controlled laboratory conditions, single soil type), the discussion is superficial. The authors mention exploring "mango, papaya, watermelon" as alternative fruit sources but do not discuss other critical limitations such as seasonal variability in fruit peel composition, scaling challenges, or regulatory considerations for agricultural use.

16- Page 6-7, Lines 140-143: The manuscript does not characterize the crosslink density, network structure, or gel fraction of the synthesized hydrogels. These parameters are fundamental to understanding hydrogel properties and are typically reported for hydrogel studies. Without this information, it is impossible to assess whether the crosslinking was successful or to predict hydrogel behavior.

17- Page 17, Lines 371-377: The manuscript proposes using fruit peel-derived hydrogels in agricultural systems but does not address regulatory approval, food safety, or potential toxicity concerns. If these hydrogels are used in food crop production, regulatory agencies may require safety assessments. The presence of residual extraction chemicals or microbial contaminants is not discussed.

18- Page 8, Lines 166-171: The manuscript reports final swelling ratios but does not analyze swelling kinetics. The time-dependent swelling data are presented in Figure 2 but not discussed in detail. The authors do not report swelling half-times, maximum swelling rates, or whether swelling follows predictable kinetic models (e.g., Fickian or anomalous diffusion).

19- Page 14-16, Lines 285-336: The manuscript presents extensive characterization data (SEM, EDX, FTIR) but fails to establish clear correlations between structural properties and functional performance (swelling, water retention, degradation). For example, the relationship between pore size (from SEM) and swelling capacity is not discussed. The role of functional groups (from FTIR) in water absorption is not analyzed.

20- Page 3-4, Lines 52-88: The manuscript does not reference or discuss recent advances in sustainable hydrogel materials and molecular reconstruction techniques. Recent publications on advanced hydrogel synthesis and property enhancement are not cited, limiting the contextual positioning of this work within the broader field.

1. Li, D., Ning, S., Yu, L., Jiang, F., Zhao, D., Zhang, S.,... Li, L. (2025). Molecular Reconstruction for the High-Performance Recycled Fluororubbers. Advanced Materials, 37(24), 2501622. doi: https://doi.org/10.1002/adma.202501622

2. Long, Q., Jiang, G., Zhou, J., Zhao, D., & Yu, H. (2024). A Cellulose Ionogel with Rubber-Like Stretchability for Low-Grade Heat Harvesting. Research, 7, 533. doi: 10.34133/research.0533

Reviewer #2: COMMENTS FOR MANUSCRIPT PONE-D-26-00016

Sustainable Hydrogel from Biowaste: Synthesis and Characterization of Pectin and

Starch-Based Hydrogels Derived from Fruit Peel

Major Revision-

1. Similar pectin/starch hydrogels from fruit waste for swelling and soil retention already exist in literature. A) https://doi.org/10.24191/srj.v22i1.17308 Preparation and Characterization of Pectin-Starch Hydrogel Modified with Urea and Lactic Acid-Based Deep Eutectic Solvent for Soil Water Retention

b) https://doi.org/10.3390/cleantechnol6010001- From Fruit Waste to Hydrogels for Agricultural Applications

Emphasize Gulf region novelty and highlight heavy metal adsorption as a unique contribution. Add Introduction. Reframe the discussion as the “first comprehensive Gulf study” and include a comparison table against the literature, which has a 65-75% topical overlap. Strengthen regional specificity for arid agriculture

2. Extraction and synthesis were performed under fixed conditions without statistical optimization (e.g., RSM or factorial design). Please justify parameter selection (e.g., preliminary trials or literature basis). Consider framing this as a baseline study and note in the discussion that optimization is recommended for future scale-up.

3. While pot experiments demonstrate improved water retention, plant growth indicators (e.g., germination, biomass, yield) are not included, limiting support for agricultural performance claims. The authors have already acknowledged this limitation in the discussion by noting controlled conditions, single soil type, and the need for broader climatic evaluation. This acknowledgement is appropriate. However, no additional experiments are required but ensure that claims throughout the manuscript remain appropriately moderated and consistent with the stated limitations.

4. Swelling behaviour was evaluated only in distilled water, which reduces applicability to saline or arid soils. Indicate this more explicitly as a limitation and recommend future testing under ionic or saline conditions representative of arid soils.

5. Consider including inferential statistics (e.g., ANOVA or t-tests) where applicable to support comparisons between hydrogel formulations and treatments.

Minor Revisions

Methods

• Specify banana ripeness stage, as starch yield is maturity-dependent in Lines 100-101.

• Provide complete soil characterization (pH, texture, moisture-holding capacity) in Lines 172–177.

• Recheck Lines 118–189 for clarity and consistency.

Results

• Verify the reference in Line 222.

• Clarify what 250 min represents (equilibrium time or t₉₀)?

• Include statistical significance for water-retention comparisons.

• Rephrase lines 299 and 320 for clarity.

Writing and Presentation

• The wording in Lines 93-98 is slightly inconsistent. Line 93 frames the work as a hypothesis, whereas Line 95 states that the study reports outcomes, which reads as conclusive. For clarity and a more conventional scientific tone, consider using a consistent objective-driven phrasing.

• Consider revising the title to reflect agricultural application. The paper states that hydrogels are made for agriculture, but the title doesn’t mention this.

• Units missing, for example, “2.16 pectin" → "2.16% pectin.”

• The pectin extraction was performed at a single fixed condition (pH 1.5, 90 °C) without optimization or justification; please provide a rationale or clarify that this represents baseline rather than optimized parameters like OFAT/OVAT as mentioned above in point 1.

• Since validation only utilized commercial potting soil, claims of UAE or Gulf desert applicability are not completely justified; consider modifying the claim or admitting the need for testing on representative soils (such as saline or sandy).

• Only post-burial EDX detection presently supports the adsorption claim; quantitative adsorption studies (capacity, kinetics, isotherms) or a toned-down claim is advised.

• Correct Figure 1 reference error (line 118)

Overall recommendation

Accept after moderate revisions

The study presents solid experimental work and contributes to sustainable hydrogel development from biowaste. However, clearer positioning relative to existing literature, improved methodological justification, moderated regional claims, and minor analytical clarifications are needed to strengthen the manuscript prior to publication.

**********

6. PLOS authors have the option to publish the peer review history of their article (what does this mean?). If published, this will include your full peer review and any attached files.

If you choose “no”, your identity will remain anonymous but your review may still be made public.

**Do you want your identity to be public for this peer review?**  For information about this choice, including consent withdrawal, please see our Privacy Policy.

Reviewer #1: No

Reviewer #2: No

**********

[NOTE: If reviewer comments were submitted as an attachment file, they will be attached to this email and accessible via the submission site. Please log into your account, locate the manuscript record, and check for the action link "View Attachments". If this link does not appear, there are no attachment files.]

To ensure your figures meet our technical requirements, please review our figure guidelines: https://journals.plos.org/plosone/s/figures

You may also use PLOS’s free figure tool, NAAS, to help you prepare publication quality figures: https://journals.plos.org/plosone/s/figures#loc-tools-for-figure-preparation.

NAAS will assess whether your figures meet our technical requirements by comparing each figure against our figure specifications.

Attachment Submitted filename: Reviewer_Comments_PONE-D-26-00016.docx

# Author comment: Author response to Decision Letter 1

**DOI:** 10.1371/journal.pone.0354244.r002

21 Apr 2026

We sincerely thank the Academic Editor and Reviewers for their constructive and insightful comments. We have carefully revised the manuscript to address all concerns. All changes have been incorporated and highlighted in the revised version. A detailed point-by-point response is provided in the attached separate file "Response to Reviewers".

Attachment Submitted filename: Response to Reviewers.docx

# Peer review: Decision Letter 1

**DOI:** 10.1371/journal.pone.0354244.r003

**Authors:** Pankaj Tomar

6 Jul 2026

Sustainable Hydrogel from Biowaste: Synthesis and Characterization of Pectin and Starch-Based Hydrogels Derived from Fruit Peel

PONE-D-26-00016R1

Dear Author

We’re pleased to inform you that your manuscript has been judged scientifically suitable for publication and will be formally accepted for publication once it meets all outstanding technical requirement,

Kind regards,

Dr. Pankaj TOMAR

Academic Editor

PLOS One

Additional Editor Comments (optional):

Reviewers' comments:

Reviewer's Responses to Questions

**Comments to the Author**

1. If the authors have adequately addressed your comments raised in a previous round of review and you feel that this manuscript is now acceptable for publication, you may indicate that here to bypass the “Comments to the Author” section, enter your conflict of interest statement in the “Confidential to Editor” section, and submit your "Accept" recommendation.

Reviewer #2: All comments have been addressed

Reviewer #3: All comments have been addressed

**********

2. Is the manuscript technically sound, and do the data support the conclusions?

The manuscript must describe a technically sound piece of scientific research with data that supports the conclusions. Experiments must have been conducted rigorously, with appropriate controls, replication, and sample sizes. The conclusions must be drawn appropriately based on the data presented.

Reviewer #2: Partly

Reviewer #3: Yes

**********

3. Has the statistical analysis been performed appropriately and rigorously?

Reviewer #2: Yes

Reviewer #3: Yes

**********

4. Have the authors made all data underlying the findings in their manuscript fully available?

The PLOS Data policy requires authors to make all data underlying the findings described in their manuscript fully available without restriction, with rare exception (please refer to the Data Availability Statement in the manuscript PDF file). The data should be provided as part of the manuscript or its supporting information, or deposited to a public repository. For example, in addition to summary statistics, the data points behind means, medians and variance measures should be available. If there are restrictions on publicly sharing data—e.g. participant privacy or use of data from a third party—those must be specified.

Reviewer #2: Yes

Reviewer #3: Yes

**********

5. Is the manuscript presented in an intelligible fashion and written in standard English?

PLOS ONE does not copyedit accepted manuscripts, so the language in submitted articles must be clear, correct, and unambiguous. Any typographical or grammatical errors should be corrected at revision, so please note any specific errors here.

Reviewer #2: Yes

Reviewer #3: Yes

**********

6. Review Comments to the Author

Please use the space provided to explain your answers to the questions above. You may also include additional comments for the author, including concerns about dual publication, research ethics, or publication ethics. (Please upload your review as an attachment if it exceeds 20,000 characters)

Reviewer #2: I thank the authors for their careful and detailed responses to the reviewers' comments. The revised manuscript has been substantially improved. The authors have appropriately clarified the novelty and scope of the study, justified the methodological choices, moderated the claims regarding agricultural applicability, incorporated statistical analyses, and acknowledged the limitations related to saline conditions, field validation, and adsorption quantification.

Although quantitative adsorption studies and field-scale evaluations remain subjects for future work, the authors have appropriately framed the present study as a baseline material development and functional validation study. The remaining issues are minor and do not affect the overall scientific quality of the manuscript.

Reviewer #3: The manuscript presents a well-executed and scientifically sound study with clear methodology, comprehensive analysis, and well-supported conclusions. The results are presented logically and make a meaningful contribution to the field. The manuscript is well organized, and I found no significant technical, ethical, or publication concerns. Overall, the work meets the journal's standards and is suitable for publication. I recommend acceptance in its present form.

**********

7. PLOS authors have the option to publish the peer review history of their article (what does this mean?). If published, this will include your full peer review and any attached files.

If you choose “no”, your identity will remain anonymous but your review may still be made public.

**Do you want your identity to be public for this peer review?**  For information about this choice, including consent withdrawal, please see our Privacy Policy.

Reviewer #2: No

Reviewer #3: No

**********

# Editor report: Acceptance letter

**DOI:** 10.1371/journal.pone.0354244.r004

**Authors:** Pankaj Tomar

PONE-D-26-00016R1

PLOS One

Dear Dr. KHAN,

I'm pleased to inform you that your manuscript has been deemed suitable for publication in PLOS One. Congratulations! Your manuscript is now being handed over to our production team.

At this stage, our production department will prepare your paper for publication. This includes ensuring the following:

* All references, tables, and figures are properly cited

* All relevant supporting information is included in the manuscript submission,

* There are no issues that prevent the paper from being properly typeset

You will receive further instructions from the production team, including instructions on how to review your proof when it is ready. Please keep in mind that we are working through a large volume of accepted articles, so please give us a few days to review your paper and let you know the next and final steps.

Lastly, if your institution or institutions have a press office, please let them know about your upcoming paper now to help maximize its impact. If they'll be preparing press materials, please inform our press team within the next 48 hours. Your manuscript will remain under strict press embargo until 2 pm Eastern Time on the date of publication. For more information, please contact onepress@plos.org.

You will receive an invoice from PLOS for your publication fee after your manuscript has reached the completed accept phase. If you receive an email requesting payment before acceptance or for any other service, this may be a phishing scheme. Learn how to identify phishing emails and protect your accounts at https://explore.plos.org/phishing.

If we can help with anything else, please email us at customercare@plos.org.

Thank you for submitting your work to PLOS ONE and supporting open access.

Kind regards,

PLOS ONE Editorial Office Staff

on behalf of

Dr. Pankaj Tomar

Academic Editor

PLOS One
